# Discovery of a potent covalent inhibitor that unusually distorts the catalytic dyad of SARS-CoV-2 main protease

**DOI:** 10.1128/jvi.00658-25

**Published:** 2025-09-15

**Authors:** Juan Wang, Xiaohong Sang, Wenyan Zheng, Jasper Fuk-Woo Chan, Jiao Zhou, Yan Xu, Pu Han, Yong Feng, Lifeng Fu, Jessica Oi-Ling Tsang, Shuofeng Yuan, Aaron Ciechanover, Jing An, Kwok-Yung Yuen, Jianxun Qi, Ziwei Huang

**Affiliations:** 1 School of Life Sciences, Tsinghua University 12442, Beijing, China; 2 Ciechanover Institute of Precision and Regenerative Medicine, School of Medicine, The Chinese University of Hong Kong 407605, Shenzhen, China; 3 State Key Laboratory for Conservation and Utilization of Subtropical Agro-Bioresources, Guangxi University 12664, Nanning, China; 4 CAS Key Laboratory of Pathogen Microbiology and Immunology, Institute of Microbiology, Chinese Academy of Sciences 85387, Beijing, China; 5 State Key Laboratory of Emerging Infectious Diseases, Department of Microbiology, School of Clinical Medicine, Li Ka Shing Faculty of Medicine, The University of Hong Kong, Pokfulam, Hong Kong Special Administrative Region, China; 6 InnoHK (Centre for Virology, Vaccinology and Therapeutics), Hong Kong Science and Technology Park 646266 , Hong Kong Special Administrative Region, China; 7 Department of Infectious Diseases and Microbiology, The University of Hong Kong-Shenzhen Hospital 444333, Shenzhen, Guangdong Province, China; 8 Pandemic Research Alliance Unit, The University of Hong Kong, Hong Kong Special Administrative Region, China; 9 Guangzhou Laboratory, Guangzhou, Guangdong, China; 10 Department of Microbiology, Queen Mary Hospital, Pokfulam, Hong Kong Special Administrative Region, China; 11 The Chinese University of Hong Kong, Shenzhen Futian Biomedical Innovation R&D Center 407605, Shenzhen, China; 12 Division of Infectious Diseases and Global Public Health, Department of Medicine, School of Medicine, University of California at San Diego 8784, La Jolla, California, USA; 13 Technion Rappaport Integrated Cancer Center, The Rappaport Faculty of Medicine and Research Institute, Technion Israel Institute of Technology 26747, Haifa, Israel; St Jude Children's Research Hospital, Memphis, Tennessee, USA

**Keywords:** SARS-CoV-2, main protease, covalent inhibitor, catalytic dyad, biochemical probe

## Abstract

Proteases are versatile therapeutic targets for a wide variety of human diseases, including cancer, cardiovascular diseases, and infections by viruses, bacteria, and parasites. The main protease (M^pro^) of severe acute respiratory syndrome coronavirus 2 (SARS-CoV-2), the virus that causes coronavirus disease 2019 (COVID-19), has been one of the major drug targets for the treatment of COVID-19. In this study, a small-molecule covalent inhibitor, **H102**, of SARS-CoV-2 M^pro^ was developed from serial structural modifications on a compound previously reported by us to have anti-SARS-CoV-2 activity. **H102** exhibited very high potency in SARS-CoV-2 M^pro^ inhibition with IC_50_ of 8.8 nM and strongly prevented SARS-CoV-2 replication in VeroE6 cells. The co-crystal structure of **H102** bound to M^pro^ determined at 1.50 Å resolution provided a structural mechanism of **H102**’s action and revealed an unusual distortion of the catalytic dyad of the viral enzyme which was caused by the benzyl ring of P2 position of **H102** interacting with the side chain of catalytic dyad His41 residue of M^pro^ and pulling His41 side chain away from the other catalytic dyad Cys145 residue. This structural mechanism of **H102** is very different from that of other reported covalent M^pro^ inhibitors for which His41 side chain orientation and its close proximity to Cys145 remain unchanged with the binding of a covalent inhibitor. As such, **H102** may serve as a biochemical probe for investigating further an unusual mechanism of the viral enzyme’s catalytic dyad disruption and inhibition, and as a prototype distinct from other reported covalent inhibitors for the development of novel antiviral agents.

**IMPORTANCE:**

A nanomolar potent small-molecule inhibitor, **H102**, of SARS-CoV-2 M^pro^ was developed and exhibited strong anti-SARS-CoV-2 infection activity in cells. Co-crystal structure determination of its complex with M^pro^ provided a structural mechanism of **H102**’s action and revealed an interesting structural feature: the benzyl ring at the P2 position of **H102** interacts with the reorientated His41 side chain, accompanied by a significant increase of the distance between the catalytic dyad Cys145-His41 residues, which is uncommon in reported covalent inhibitors. Compound **H102** may be used as a biochemical probe to further investigate mechanisms of M^pro^ inhibition and potentially different type of lead for developing antiviral agents for treating disease caused by novel coronavirus SARS-CoV-2.

## INTRODUCTION

Proteases are found in many different viruses, including the severe acute respiratory syndrome coronavirus 2 (SARS-CoV-2) that causes the coronavirus disease 2019 (COVID-19) pandemic (1). These viral proteases are responsible for cleaving the viral precursor polyproteins at specific sites into structural proteins and functional proteins crucial for viral replication (2, 3).

The main protease (M^pro^) of SARS-CoV-2, also known as 3-chymotrypsin-like (3CL) protease, is a cysteine protease essential for viral replication and one of the major therapeutic targets for treating COVID-19 (4, 5). Nirmatrelvir (PF-07321332), a SARS-CoV-2 M^pro^ inhibitor developed by Pfizer (6), has been approved clinically to treat COVID-19 in combination with Ritonavir. Many other small-molecule inhibitors and degraders of SARS-CoV-2 M^pro^ have also been reported and are under development (5, 7–14), in addition to other targeting and intervention strategies (15).

Previously, we reported the discovery of compound 17 (16), a small-molecule inhibitor of SARS-CoV-2 infection from screening a panel of α-ketoamide analogs using cell viability and plaque reduction assays. Here, in this study, compound 17 was used as the starting template to design and synthesize a series of analogs containing modifications at various moieties of the compound including its warhead. This led to the development of a covalent inhibitor **H102**, an aldehyde analog possessing potent activities in inhibiting M^pro^ enzyme function and SARS-CoV-2 infection in cells. Co-crystal structure of **H102** bound to M^pro^ was determined at 1.50 Å resolution, which revealed very interesting information about the structural mechanism of this novel inhibitor compared to other reported covalent or noncovalent inhibitors.

## RESULTS

### Development of a nanomolar potent M^pro^ inhibitor H102

The previously reported compound 17 (16) with modest M^pro^ inhibitory potency (~45% inhibition at 100 µM) was used as a starting template for synthetic efforts to develop more potent inhibitory analogs. This led to the identification of **H96**, which contains substitutions at the Cap, P1 (a six-membered lactam Gln mimic), and warhead positions (Table 1). **H96** showed over a 10-fold increase in M^pro^ inhibitory potency (IC_50_ of 9.4 µM) compared to compound 17.

**TABLE 1:** The M^pro^ inhibitory potency of compounds with modifications at the Cap, P2, P3, and warhead

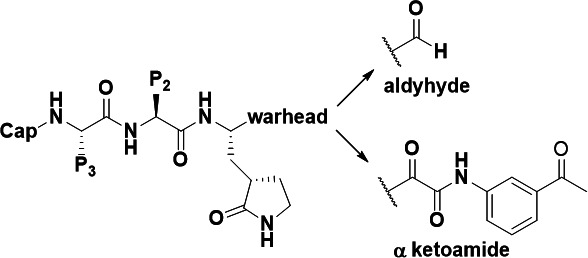					
Compd.^*a*^	Cap	P_3_	P_2_	Warhead	M^pro^ IC_50_
H96	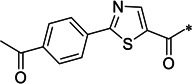	CH_3_	(4-CH_3_O)-Ph	Ketoamide	9.4 ± 3.0 µM
H94	Boc	CH_3_	(4-CH_3_O)-Ph	Ketoamide	24.8 ± 2.3 µM
H97	Boc	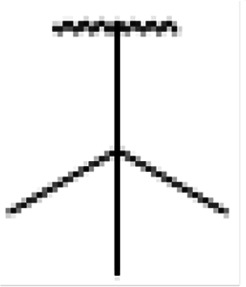	(4-CH_3_O)-Ph	Ketoamide	6.3 ± 2.7 µM
H98	Boc	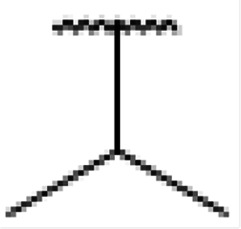	(4-CH_3_O)-Ph	Ketoamide	8.7 ± 2.5 µM
H99	Boc	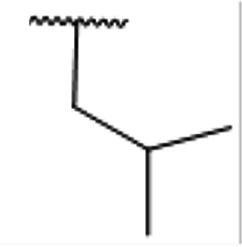	(4-CH_3_O)-Ph	Ketoamide	9.9 ± 4.7 µM
H137	Boc	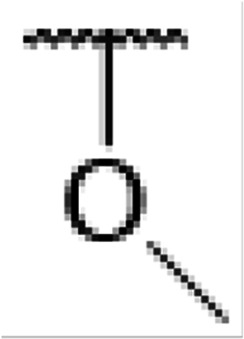	(4-CH_3_O)-Ph	Ketoamide	28.9 ± 3.8 µM
H100	Boc	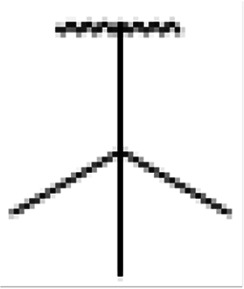	Ph	Ketoamide	476.0 ± 20.0 nM
H101	Boc	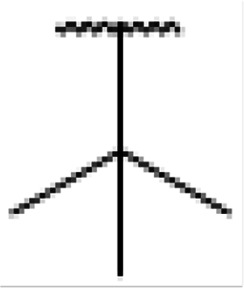	*i*-Pr	Ketoamide	3.8 ± 0.4 µM
H102	Boc	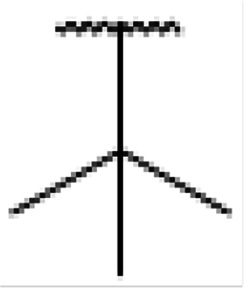	Ph	Aldehyde	8.8 ± 0.1 nM
GC376			*i*-Pr	Aldehyde	24.5 ± 2.2 nM

^*a*^ Cap and P3 of GC376 were not applicable for the formula. For the chemical structure of GC376, see reference 17.

**Fig 1:**
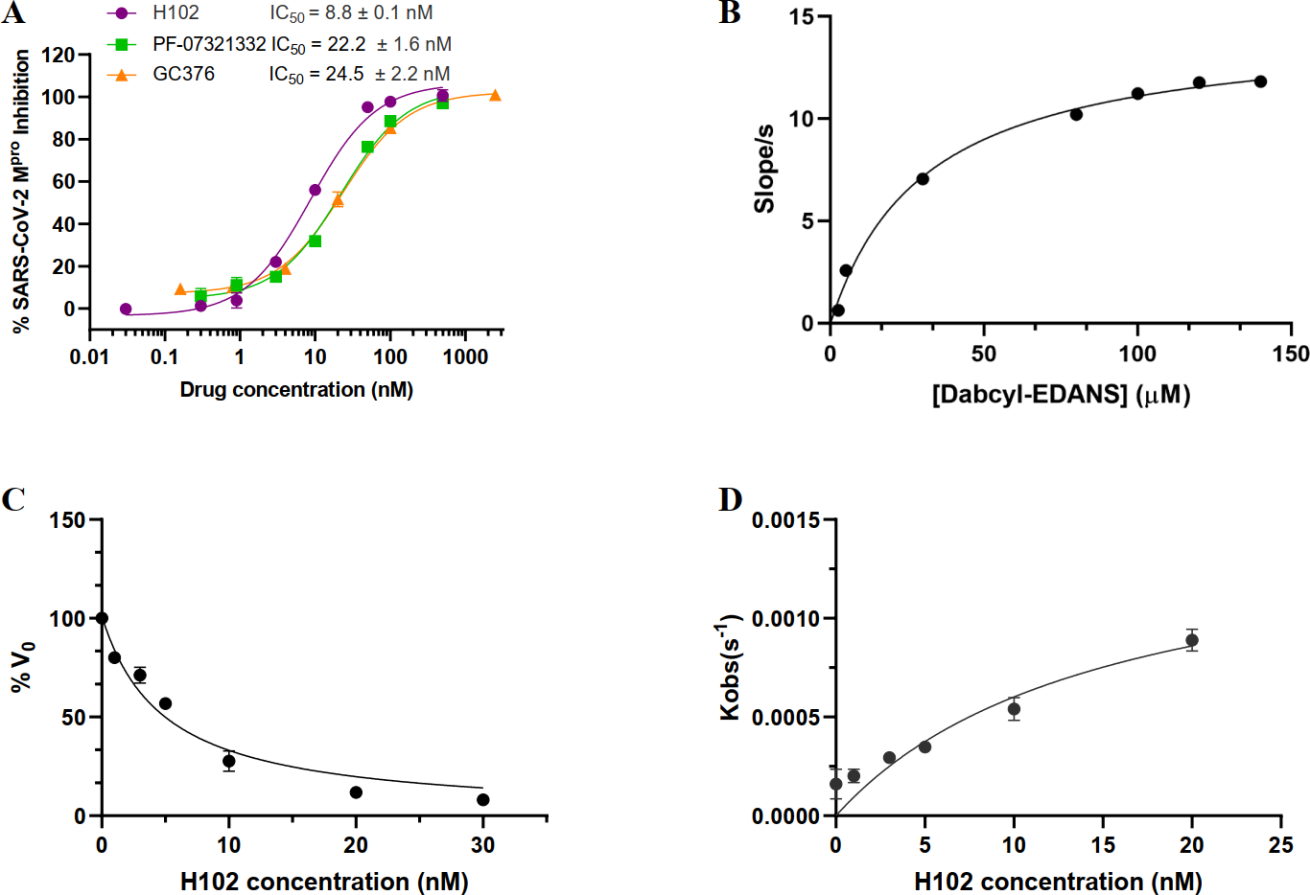
(A) Inhibition of SARS-CoV-2 M^pro^ by H102, PF-07321332, and GC376 at various final concentrations in enzyme assay as plotted on the x-axis. FRET-based peptide substrate (Dabcyl)-KTSAVLQSGFRKM-Glu(EDANS)-NH_2_ was applied as substrate. (**B**) *K*_*m*_ value of substrate Dabcyl-KTSAVLQSGFRKME-Edans for purified SARS-CoV-2 M^pro^ was determined at seven different concentrations, ranging from 2.5 to 140 µM, using Michaelis–Menten plot. (**C**) The *K*_*i*_ value of H102 was calculated from duplicate measurements by nonlinear regression using Morrison *K*_*i*_ equation (*Y* = *V*_*0*_ × (1 − ((((*E*_*t*_ + *X* + (*K*_*i*_ × (1 + (*S*/*K*_*m*_)))) − (((*E*_*t*_ + *X* + (*K*_*i*_ × (1 + (*S*/*K*_*m*_))))^2) − 4 × *E*_*t*_ × *X*)^0.5))/(2 × *E*_*t*_)))). (**D**) The *k*_inac_ and *K*_*I*_ values of H102 were calculated from duplicate measurements by nonlinear regression using equation 1: [*P*] = *V*_*i*_× (1 − exp (*−k*obs × *t*)) / *k*obs + *d* and equation 2: *k*obs = (*k*_inac_ × [I]) / ([I] + *K*_I_ × (1 + [*S*]/*K*_*m*_)).

We changed the Cap of **H96** to Boc, resulting in **H94**, which showed weaker inhibition of SARS-CoV-2 M^pro^ than **H96** (Table 1). Nevertheless, **H94** was used for further structural optimization because its structure was more amenable to synthesis than **H96**. Different side chain substitutions were incorporated at the P3 position of **H94**, resulting in **H97–99** and **H137** with the substituent tert-Leucine (Tle) for **H97** yielding the best effect (Table 1). Thus, this substituent was kept for the P3 position, while we attempted two different P2 modifications, as in **H100** and **H101**, finding that the P2-Phe in **H100** was better. Finally, we changed the warhead of **H100** from ketoamide to aldehyde while keeping the above-described P2-Phe and P3-Tle with better or best effect. This led to our final lead compound **H102** displaying the most potent M^pro^ inhibition among all analogs (IC_50_ of 8.8 nM, Table 1). **H102** was more potent than another reported aldehyde M^pro^ inhibitor GC376 (IC_50_ of 24.5 nM) and Nirmatrelvir (PF-07321332, IC_50_ of 22.2 nM), the clinically approved M^pro^ inhibitor drug by Pfizer (6), in M^pro^ inhibition assays (Fig. 1A).

### Kinetic and enzyme inhibition characterization of H102

For further kinetic and enzyme inhibition characterization studies of H102 identified from our screening study as described above, we expressed and purified the SARS-CoV-2 M^pro^ protein in *E. coli* as reported previously (18). The *K*_*m*_ value of Dabcyl-KTSAVLQSGFRKME-Edans for SARS-CoV-2 M^pro^ was determined to be 28.6 ± 3.1 μM (Fig. 1B), which is similar to the previously reported value 28.2 ± 3.4 µM (19). The *K*i of **H102** was determined to be 2.89 nM (Fig. 1C), which was in line with its high inhibitory potency measured by IC_50_. In order to understand the noncovalent and covalent contributions to high potency of **H102**, the noncovalent affinity (*K*_*I*_) and the reaction rate of covalent modification (*k*_inact_) were calculated. **H102** displays high noncovalent affinity (*K*_*I*_ = 6.84 nM) and fast reaction rate (*k*_inact_ = 0.0014 S^−1^), as shown in Fig. 1D. These results indicate both noncovalent and covalent contributions to the high potency of **H102**.

**Fig 2:**
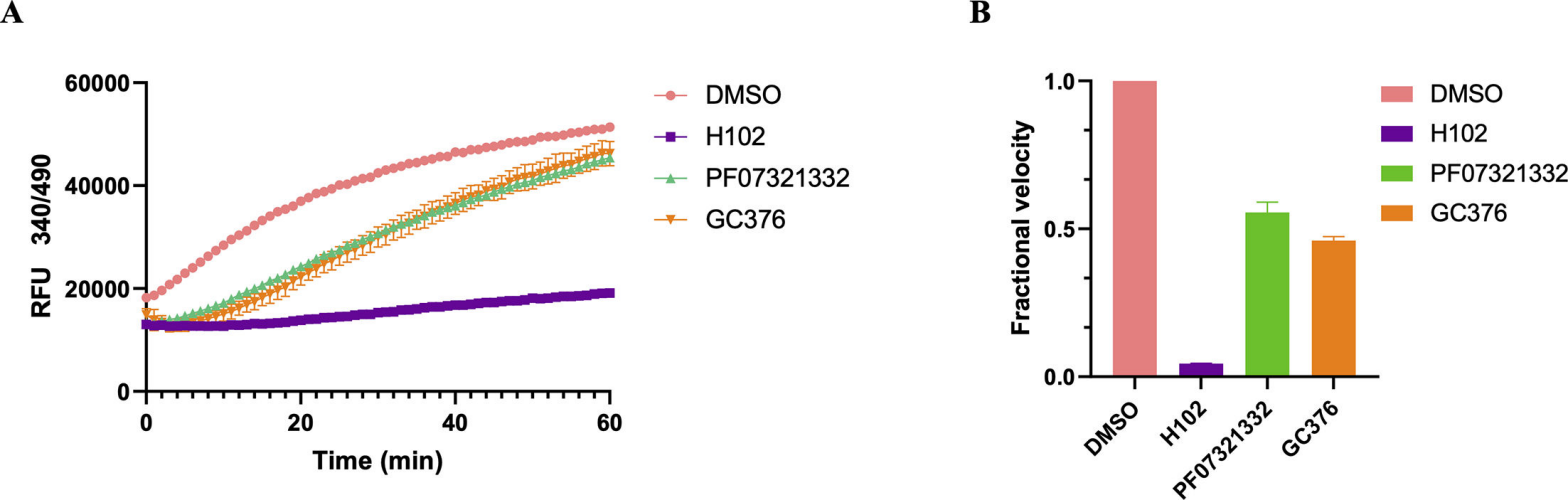
*In vitro* jump dilution experiments to evaluate the reversibility of the compound’s inhibition. (**A**) The progression curves in the presence or absence of compounds were monitored 1 h after 100-fold dilution of compound-M^pro^ complex. (**B**) The fractional velocity was determined by dividing the velocities of compound-M^pro^ complex after 100-fold dilution by the velocity with the solvent DMSO.

The recovery of M^pro^ activity indicated that both PF-07321332 and GC376 are reversible covalent M^pro^ inhibitors in jump dilution experiments (Fig. 2A). GC376 showed slower M^pro^ activity recovery velocity compared to PF-07321332 (Fig. 2B). **H102** with the same aldehyde warhead as GC376 behaved as an irreversible-characteristic inhibitor. The enzymatic activity **H102**-M^pro^ complex did not recover after 100-fold dilution within 1 h (Fig. 2A). The result suggested that a stable **H102**-M^pro^ complex was formed. The reorientation of the His41 side chain may result in a reverse reaction barrier. Therefore, **H102** displayed slower M^pro^ activity recovery velocity (Fig. 2B). These results show an interesting conversion from a reversible covalent inhibitor to an irreversible inhibitor likely due to the conformational change of a key residue.

To exclude any possible fluorescence quenching effect of **H102**, we used Fmoc-Glu–EDANS to mimic C-terminal EDANS product Ser-Gly-Phe-Arg-Lys-Met-Glu(EDANS), and the fluorescence intensity of Fmoc-Glu–EDANS solution was measured in the presence or absence of 100 nM of **H102** for 1 h. As shown in Fig. S1, the addition of **H102** showed no change or effect on the fluorescence intensity of Fmoc-Glu–EDANS in solvent (DMSO) only without the compound, demonstrating that **H102** had no fluorescence quenching effect.

### Inhibition by H102 of SARS-CoV-2 replication in cells

**Fig 3:**
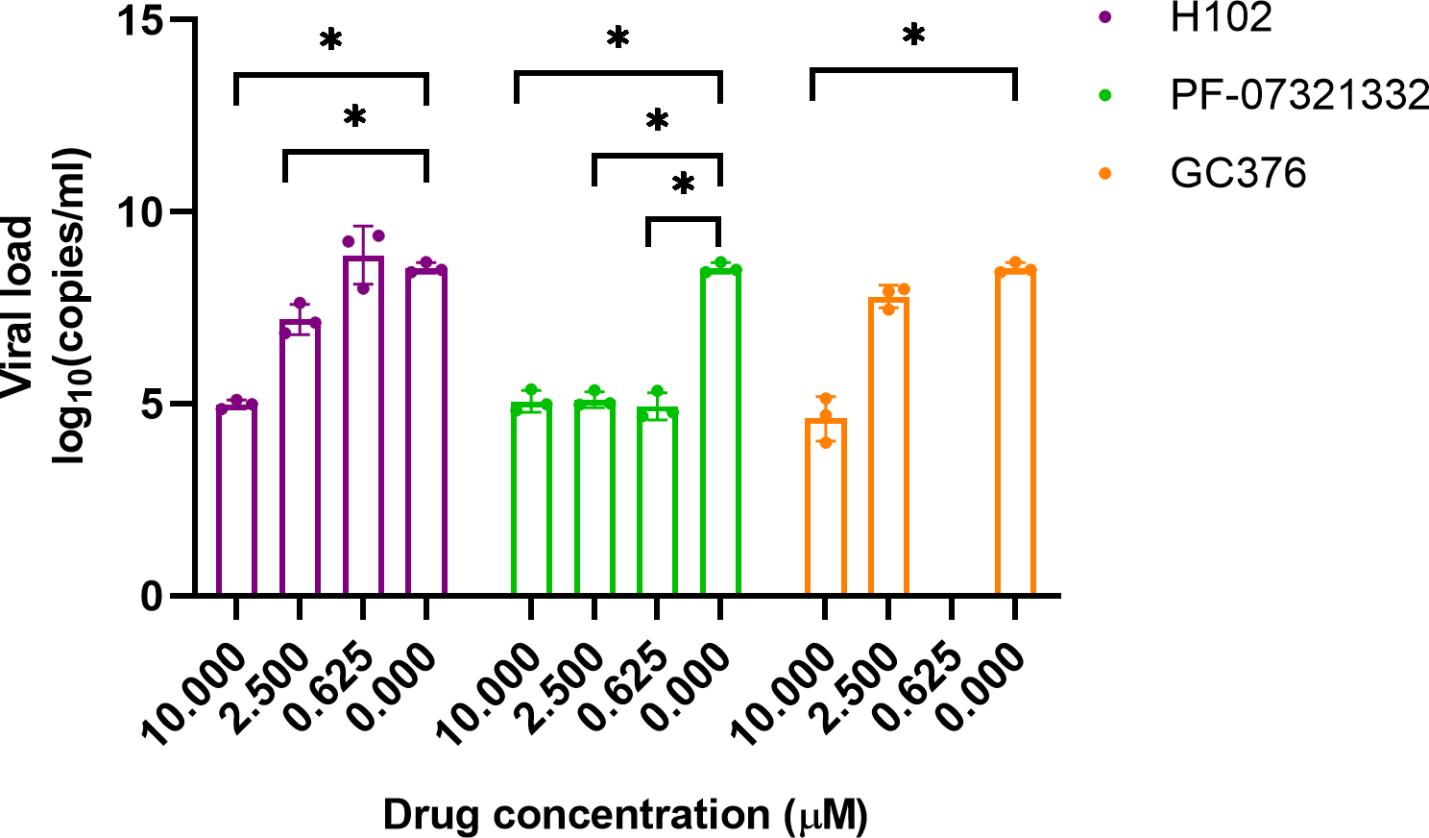
*In vitro* antiviral activity of selected compounds**.** VeroE6 cells were infected with SARS-CoV-2 (multiplicity of infection [MOI] of 0.1) and treated with various concentrations of H102, PF-07321332, GC376, or vehicle control. Viral RNA load in the cell culture supernatant samples was quantified by qRT-PCR. All experiments were performed in triplicate, and the values are presented as mean ± SD. *P*-values were calculated using Student’s *t*-test to compare each compound candidate with vehicle control. *P < 0.05.

The lead compound **H102** was examined for anti-SARS-CoV-2 activity in VeroE6 cells by viral load reduction assay. **H102** effectively decreased viral replication by 3.55 log_10_ at 10 µM and 1.34 log_10_ at 2.5 µM, respectively (Fig. 3). As controls, PF-07321332 and GC376 showed 3.48 log_10_ and 3.93 log_10_ reductions, respectively, in viral RNA load at 10 µM. **H102** inhibited viral replication with an EC_50_ of 168.9 nM in VeroE6 cells without cytotoxicity at much higher concentrations (over 50 μM, Fig. S2).

### Co-crystal structure determination of H102 in complex with SARS-CoV-2 M^pro^

**Fig 4:**
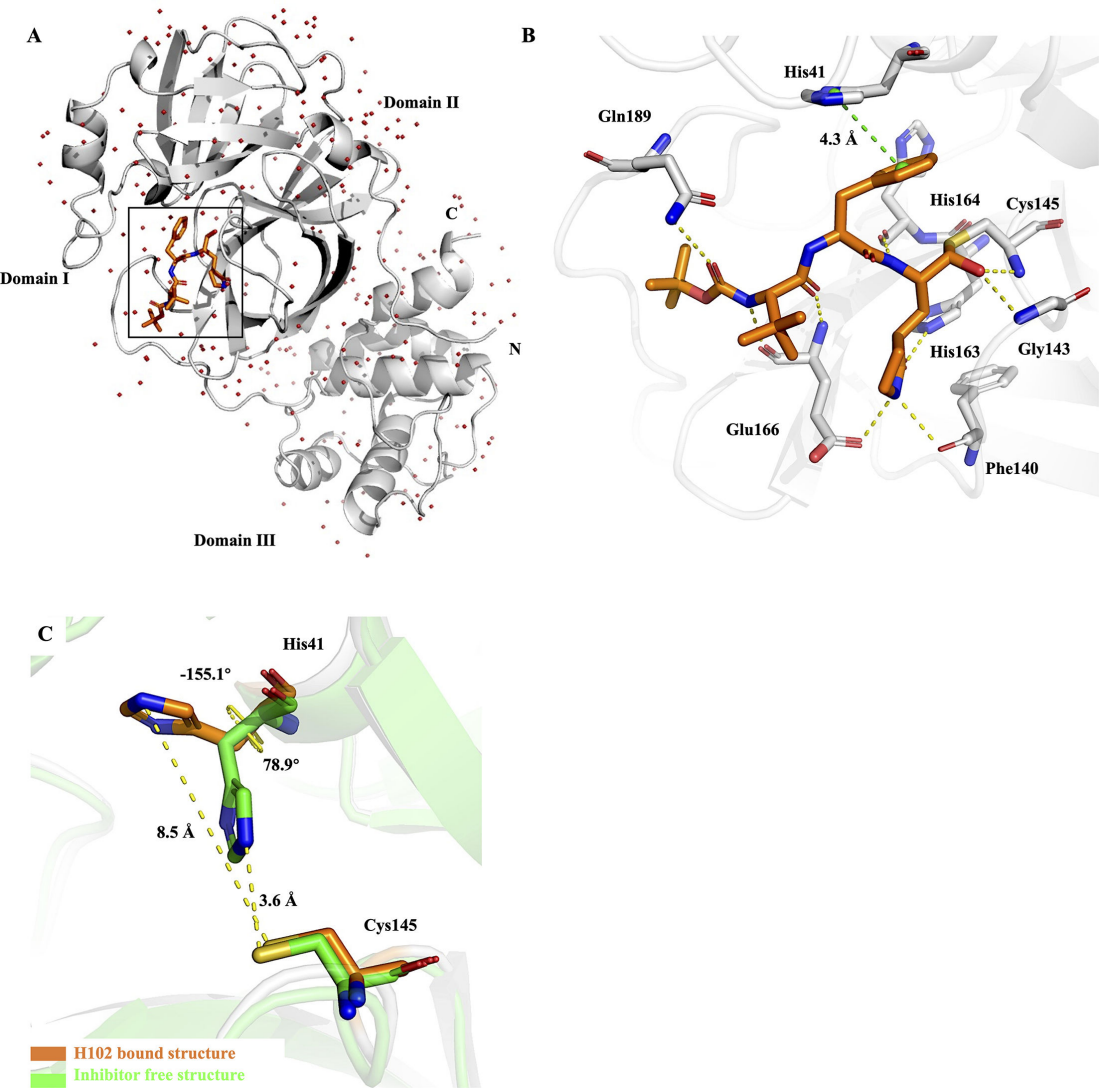
The co-crystal structure of SARS-CoV-2 M^pro^ in complex with compound H102. (**A**) Cartoon representation of the crystal structure of SARS-CoV-2 M^pro^ in complex with H102. H102 is shown as orange sticks; water molecules are shown as red spheres. (**B**) Close-up view of the H102 binding pocket. (**C**) The distances between NE2-His41 and Sγ-Cys145, and the torsion angles NCαCβCγ(χ1) of His41 are shown before H102 binding (green, PDB: 6Y2E) and after H102 binding (orange), highlighting the dramatic reorientation of His41 side chain and distortion of the geometry of catalytic dyad Cys145-His41 residues due to H102 binding.

To elucidate the mechanism of **H102** action, co-crystal structure of **H102** bound to SARS-CoV-2 M^pro^ was determined at the resolution of 1.50 Å (Fig. 4A). As revealed in this structure, the overall interactions of **H102** with SARS-CoV-2 M^pro^ are similar to those in the published co-crystal structures of other reported M^pro^ covalent inhibitors, except for the orientation of the benzyl ring at the P2 position of **H102** and the geometry of M^pro^ catalytic dyad Cys145-His41 residues (Fig. 4B) (6, 10, 20). In the complex structure of **H102** with M^pro^, the benzene ring of **H102**’s P2 position forms π-π interaction with the side chain of His41 of M^pro^ and causes His41 side chain to undergo a drastic conformational change. In the inhibitor-free structure of SARS-CoV-2 M^pro^, His41 and Cys145 constitute the catalytic dyad. The imidazole group of His41, by accepting a proton from Cys145, activates the nucleophilic attack reaction. The distance between the NE2 atom of His41 and the Sγ atom of Cys145 is 3.6 Å in the inhibitor-free structure, whereas this distance is increased to 8.5 Å after **H102** binding (Fig. 4C). The torsion angle NCαCβCγ(χ1) of His41 is 78.9° (*gauche*^−^, *g*^−^) in the inhibitor-free structure but changes to −155.1° (*trans*, t) after **H102** binding (Fig. 4C), which seems to indicate that the side chain of His41 in the inhibitor-free structure is in the least occupied conformational state because the *g*^− ^side chain conformation has a lower propensity (12.2%) than *g^+^* (54.6%) and *t* (33.2%) for the side chain conformation occurrence of His residues in proteins (21). The benzyl ring at the P2 position of **H102** is sandwiched between His41 and Cys145 (Fig. 4B), which completely distorts and blocks the catalytic dyad interactions and provides a structural basis for the highly potent inhibitory activity of **H102**.

## DISCUSSION

In this study, we attempted to optimize compound 17 previously reported by us to have anti-SARS-CoV-2 activity (16). After a series of structural modifications at various positions of the compound, including the cap, P1, P2, P3, and warhead, we were able to discover a highly potent SARS-CoV-2 M^pro^ aldehyde inhibitor **H102** whose M^pro^ inhibitory potency was improved by ~1,000-fold over the starting compound **H96**, an analog of compound 17. This demonstrated the efficacy of the structural modification strategy. Compound **H102** displayed very high potency in inhibiting M^pro^ enzymatic function (IC_50_ of 8.8 nM) and was more potent than control compounds PF-07321332 and GC376 in comparative M^pro^ inhibition assays. Furthermore, **H102** was effective in blocking SARS-CoV-2 replication in VeroE6 cells, demonstrating its utility as an antiviral agent that merits further development.

**Fig 5:**
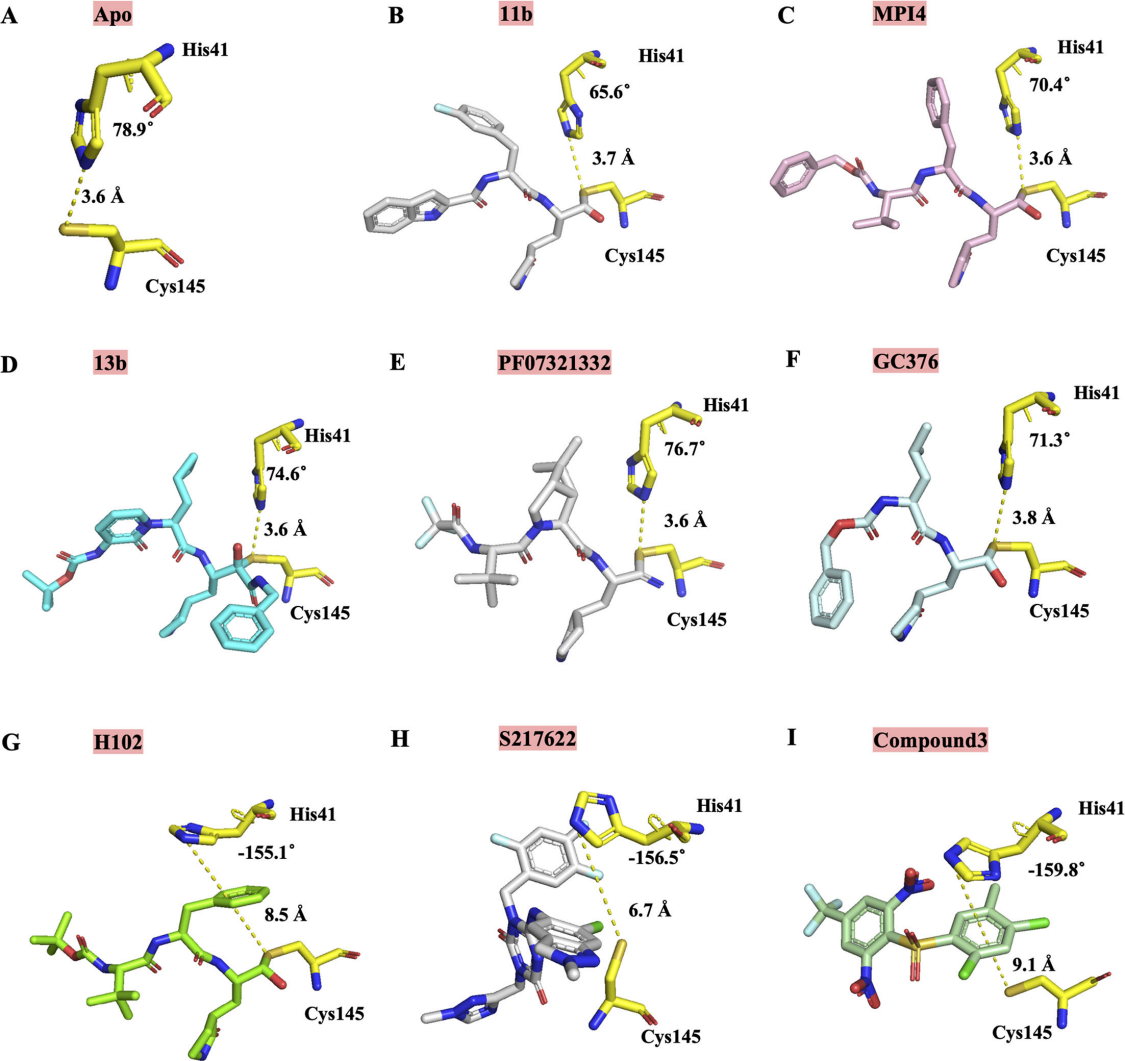
The change in the geometry of the interaction of catalytic dyad His41-Cys145 residues of M^pro^ caused by **H102** compared to the change or no or little change caused by various covalent or noncovalent inhibitors. To measure the change or lack of change, the orientation of His41 side chain and its distance to Cys145 side chain are shown in (**A**) the inhibitor-free Apo state (PDB code: 6Y2E) or when bound by covalent inhibitors (**B**) 11b (PDB code: 6M0K), (**C**) MPI4 (PDB code: 7JQ1), (**D**) 13b (PDB code: 6Y2F), (**E**) PF-07321332 (PDB code: 7VH8), (**F**) GC376 (PDB code: 7CB7), (**G**) **H102**, noncovalent inhibitors, (**H**) S-217622 (PDB code: 7VU6), and (**I**) compound 3 (PDB code: 2GZ7). The side chains of the two catalytic dyad His41-Cys145 residues are shown in yellow, while various covalent and noncovalent inhibitors are shown in different colors. The orientation of the His41 side chain is depicted by the torsion angle. The distance between His41 and Cys145 side chains is highlighted by a yellow dashed line. As shown, all other reported covalent inhibitors examined here (**B–F**) showed little or no effect in changing the orientation of His41 side chain and its distance to Cys145 side chain compared to the inhibitor-free Apo state (**A**) of M^pro^, whereas in sharp contrast, our covalent inhibitor **H102** (**G**) exerted a drastic effect in distorting the orientation of His41 side chain and its distance to Cys145 side chain, which was also observed in two noncovalent inhibitors (**H, I**).

The high-resolution co-crystal structure at 1.50 Å resolution of **H102** bound to the viral M^pro^ protein revealed an interesting mechanism of action of this inhibitor. Unlike crystal structures of other reported covalent M^pro^ inhibitors with a similar benzyl ring at P2 position, such as 11b and MPI4 (9, 20), the benzyl ring of P2 position of **H102** was projected in between the catalytic dyad of His41 and Cys145, which is different from that of 11b and MPI4 (Fig. 5B, C and G). This sandwiched interaction between **H102**’s benzyl ring and the enzyme’s catalytic dyad, as observed in the crystal structure, caused the reorientation of His41 side chain away from its inhibitor-free state and thus effectively disrupted the enzyme’s catalytic dyad geometry and function, which may structurally rationalize the strong M^pro^ inhibitory activity of **H102**. We further compared the structural mechanism of **H102** with other covalent inhibitors of M^pro^ that have other different groups at the P2 position and different warheads. As shown in Fig. 5D through F, the M^pro^ bound crystal structures of three representative covalent inhibitors including 13b (22), PF-07321332 (23), and GC376 (17), all displayed little or no distorting effect on the geometry of the catalytic dyad Cys145-His41 residues as shown by the little or no change in the orientation of His41 side chain and distance between His41 side chain and Cys145. Taken together, **H102** seems to be different from other reported covalent inhibitors in the unusual and significant distortion of the orientation of His41 side chain and geometry of Cys145-His41 interaction of viral enzyme’s catalytic dyad unseen in other covalent inhibitors.

Whether the above-described distortion of the catalytic dyad is implicated in the enzyme’s functional mechanism awaits further investigation. From other published studies in the literature, conformational variation or flexibility of an enzyme’s catalytic residues is commonly observed. About two-thirds of catalytic centers are flexible to perform enzyme functions, based on analysis of more than 60,000 protein structures in the PDB database (24). Specifically for SARS-CoV-2 M^pro^, since it cleaves polyproteins pp1a and pp1b at 11 distinct sites, this requires that the catalytic pocket of M^pro^ has sufficient flexibility to accommodate various substrates. Most M^pro^ substrates have small-sized Leu and Val in their P2 position, whereas one of the substrates has a bulky Phe in the P2 position (25). Recent studies using dynamical nonequilibrium MD simulations suggested that the S2 subpocket of M^pro^ undergoes substantial conformational rearrangement when bound by substrates with large residues such as Phe at the P2 position (26). In light of these observations reported in the literature, the distortion of the catalytic dyad due to the conformational change of catalytic residue His41 reported in our present study is in line with the notion of the enzyme’s S2 subpocket flexibility being a part of its functional mechanism.

While the structural mechanism of **H102** appears to be uncommon among covalent inhibitors of M^pro^ as described above, similar observations have been reported for noncovalent inhibitors, such as S-217622, a noncovalent oral SARS-CoV-2 M^pro^ inhibitor with IC_50_ of 13 nM (7), and compound 3, a noncovalent SARS-CoV M^pro^ inhibitor with IC_50_ of 300 nM (27). In the co-crystal structures of these noncovalent inhibitors bound to M^pro^, the 2,4,5-trifluorobenzylic moiety of S-217622 and the phenyl ring of compound 3 form face-to-face π interactions with the rotated side chain of His41, similar to the case of **H102** (Fig. 5G–I). These compounds**—H102**, S-217622, and compound 3—share a similar feature of having aromatic groups interacting favorably with a positively charged His41 side chain. This interaction may facilitate the His41 side chain to cross the energy barrier from the less occurring *g*^−^ conformational state (in the inhibitor-free structure) to the more occurring *t* state (inhibitor-bound structure), thus allowing the observed conformational change to occur. Whether this interesting structural mechanism shared by one covalent inhibitor reported here and two noncovalent inhibitors recently reported by others could be exploited for M^pro^ inhibitor design remains to be further investigated. It is intriguing to postulate that the two noncovalent inhibitors discussed here may take advantage of their distorting effect on the geometry of catalytic dyad Cys145-His41 distance and interaction for more effective blockade of the enzyme function. On the other hand, covalent inhibitors devoid of such a distorting effect on the catalytic dyad, except for the rare case of **H102** reported here, use covalent bond formation with Cys145 to gain binding free energy and disable Cys145’s catalytic role. Would it be possible that **H102**, capable of both of these two inhibition mechanisms adopted by noncovalent and covalent inhibitors, respectively, suggests a new and different prototype of more advantageous inhibitors? This possibility seems to be supported by a recently published study by others of the design of SARS-CoV-2 M^pro^ inhibitors based on the concept of dual inhibition (i.e., disrupting the catalytic dyad’s His41 while covalently inhibiting Cys145) (28), which is reminiscent of the finding and notion described in our study here.

### Conclusion

A nanomolar potent small-molecule inhibitor, **H102**, of SARS-CoV-2 M^pro^ was developed from serial structural modifications starting from our previously reported anti-SARS-CoV-2 compound 17 (16). Compound **H102** exhibited strong anti-SARS-CoV-2 infection activity in cells. Co-crystal structure determination of its complex with M^pro^ provided a structural mechanism of **H102**’s action and revealed an interesting structural feature involving the benzyl ring of P2 position of **H102** interacting with the reorientated His41 side chain and significant increase of the distance between the catalytic dyad Cys145-His41 residues which is uncommon in reported covalent inhibitors. Compound **H102** may be used as a biochemical probe to further investigate mechanisms of M^pro^ inhibition and potentially different type of lead for developing antiviral agents for treating disease caused by novel coronavirus SARS-CoV-2.

## MATERIALS AND METHODS

### Compound synthesis and characterization

The details of synthetic methods for preparing target compounds, as well as ^1^H NMR and ^13^C NMR spectra of intermediates and target compounds, are provided in the supplemental material.

### Protein expression and purification of SARS-CoV-2 M^pro^

The pET-28b-SARS-CoV-2 M^pro^ plasmid was transformed into *E. coli* strain BL21(DE3) cells and then cultured in LB medium containing 50 µg/mL kanamycin in a shaking incubator at 37°C. When the cells were grown to an OD_600_ of 0.6–0.8, 0.6 mM IPTG was added to the cell culture to induce the protein expression at 16°C. After 18 h, the cells were harvested by centrifugation at 4,000 rpm for 20 min at 4°C. The cell pellets were washed twice by PBS, resuspended in lysis buffer (50 mM HEPES, 300 mM NaCl, 10 mM imidazole, pH 7.5), and lysed by sonication on ice using 3-second ON/5-second OFF cycles for a total of 30 min. The lysate was then clarified by ultracentrifugation at 18,000 rpm at 4°C for 40 min to remove debris. The supernatants were then purified by TALON metal affinity resin (TaKaRa, 635501) and washed with washing buffer (25 mM HEPES, 500 mM NaCl, pH 7.5) to remove unspecific binding proteins. The SUMO-His-tagged SARS-CoV-2 M^pro^ was eluted by elution buffer (25 mM HEPES, 500 mM NaCl, 300 mM imidazole, pH7.5). The SUMO-His-tagged SARS-CoV-2 M^pro^ was then treated overnight at 4°C with His-tagged SUMO protease (home-made) to remove the SUMO-His-tag. The SARS-CoV-2 M^pro^ was further purified by His60 Ni superflow resin (TaKaRa, 635659). The quality of SARS-CoV-2 M^pro^ was checked by Coomassie-stained SDS-PAGE gel. The concentration was determined via BCA Protein Assay Kit. The purified SARS-CoV-2 M^pro^ was stored in (25 mM HEPES, 1 mM DTT, 1 mM EDTA, 10% glycerol, pH 7.5).

### SARS-CoV-2 M^pro^ enzyme inhibition assay

SARS-CoV-2 M^pro^ enzyme inhibition assay for the evaluation of M^pro^ inhibitory potency of compounds was performed according to a commonly used method reported by others (29, 30). Specifically, the enzyme inhibition assay was carried out in assay reaction buffer (25 mM HEPES, 1 mM DTT, 1 mM EDTA, 0.01% Triton X-100, pH 7.5) by pre-incubating 85 µL SARS-CoV-2 M^pro^ (final concentration of 150 nM in a total volume of 100 µL) with 5 µL compounds at various concentrations. For H102, the final concentration range was from 0.03 nM to 500 nM (in a total volume of 100 µL). The mixture was incubated at 37°C with gentle shaking for 30 min in blank 96-well plates. While we did not know whether our compounds to be tested had slow onset binding, we adopted the preincubation step reported by others in our enzyme inhibition assays (30, 31). Next, 10 µL of 250 µM M^pro^ fluorogenic substrate (Dabcyl-KTSAVLQSGFRKME-Edans, final concentration of 25 µM in a total volume of 100 µL) was added to the reaction mixture, after which the plate was incubated at 37°C for 1 h. The relative fluorescence units (RFU) were measured at a single time point after 1 h of incubation using a PerkinElmer EnVision multimode plate reader with an excitation wavelength of 340 nm and an emission wavelength of 490 nm. Percent inhibition was calculated based on control wells containing no compound (100% activity) and a blank control. The IC_50_ values were calculated using GraphPad Prism software. All experiments were performed in triplicate, and the values are presented as mean ± SD.

### Kinetic assay

*K*_*m*_ value for substrate Dabcyl-KTSAVLQSGFRKME-Edans was determined at seven different concentrations, ranging from 2.5 to 140 µM (19). The relative fluorescence units (RFU) were monitored continuously by addition of SARS-CoV-2 M^pro^ (final concentration of 100 nM) for 10 min. *K*_*m*_ value was obtained by nonlinear regression using the Michaelis-Menten plot. Then, 85 µL SARS-CoV-2 M^pro^ (final concentration of 10 nM in a total volume of 100 µL) was added to blank 96-well plates, and 5 µL H102 with various concentrations (final concentration in a total volume of 100 µL: 0, 1, 3, 5, 10, 20, and 30 nM) and 10 µL of 250 µM substrate (final concentration of 25 µM in a total volume of 100 µL) were added immediately. The RFU was measured every 2 min for 2 h by a PerkinElmer EnVision multimode plate reader. The *K*_*i*_ value was calculated from duplicate measurements by nonlinear regression using Morrison Ki equation (*Y* = *V*_0_ × (1 − ((((*E*_*t*_ + *X* + (*K*_*i*_ × (1 + (*S*/*K*_*m*_)))) − (((*E*_*t*_ + *X* + (*K*_*i*_ × (1 + (*S*/*K*_*m*_))))^2) − 4 × *E*_*t*_ × *X*)^0.5))/(2 × *E*_*t*_)))) (6, 32, 33).

### Reversibility assay

To evaluate the reversibility of SARS-CoV-2 M^pro^ inhibition by the compounds, SARS-CoV-2 M^pro^ at a final concentration of 1 µM was incubated with 1 µM H102, PF-07321332, GC376, or DMSO for 30 min. Then, 1 µL reaction mixture was diluted 100-fold with 99 µL reaction buffer (25 mM HEPES, 1 mM DTT, 1 mM EDTA, 0.01% Triton X-100, pH 7.5) containing fluorogenic substrate at the concentration of 25 µM in blank 96-well plates. The RFU was measured immediately every 1 min for 1 h by a PerkinElmer EnVision multimode plate reader with an excitation wavelength of 340 nm and an emission wavelength of 490 nm. The fractional velocity was determined by dividing the velocity of added compound after 100-fold dilution by the velocity with the solvent DMSO. Three independent experiments were carried out. The values are expressed as the mean ± SD.

### Fluorescence control assay

We conducted a fluorescence control experiment using Fmoc-Glu–EDANS to mimic C-terminal EDANS product Ser-Gly-Phe-Arg-Lys-Met-Glu(EDANS) with or without the compound. Briefly, 5 µL H102 (final concentration of 100 nM in a total volume of 100 µL) and solvent (DMSO) and 10 µL Fmoc-Glu–EDANS (final concentration of 25 µM in a total volume of 100 µL) were added to 85 µL reaction buffer (25 mM HEPES, 1 mM DTT, 1 mM EDTA, 0.01% Triton X-100, pH 7.5) in blank 96-well plates. The RFU was immediately measured every 1 min for 1 h by a PerkinElmer EnVision multimode plate reader with an excitation wavelength of 340 nm and an emission wavelength of 490 nm. Three independent experiments were carried out. The values are expressed as the mean ± SD.

### Cytotoxicity assay

The cytotoxicity of H102 was measured by CellTiter-Glo Luminescent Cell Viability Assay (Promega, G7570). Briefly, VeroE6 cells were seeded into 96-well plates and incubated overnight. On the second day, cells were treated with serially diluted concentrations of the H102 for 48 h. On the day of analysis, CellTiter-Glo reagents were added to induce cell lysis. After incubating at room temperature for 10 min in the dark, luminescent signal was detected using a PerkinElmer EnVision multimode plate reader. The experiments were performed in triplicate, and the values are presented as mean ± SD.

### SARS-CoV-2 viral load reduction assay

Viral load reduction assay was performed for the evaluation of antiviral activity as we described previously (34). Briefly, SARS-CoV-2-infected VeroE6 cells were treated with different concentrations of compounds or dimethyl sulfoxide (DMSO) control. Then, cell culture supernatant samples were collected at 48 h post-inoculation (hpi) for qRT-PCR analysis of viral RNA load. Culture supernatant was lysed with buffer AVL and then extracted for total RNA with the QIAamp viral RNA mini kit (Qiagen). qRT-PCR was used for quantitation of SARS-CoV-2 viral load using the QuantiNova Probe RT-PCR kit (Qiagen) with a LightCycler 480 Real-Time PCR System (Roche). Each 20 µL reaction mixture contained 10 µL of 2 × QuantiNova Probe RT-PCR Master Mix, 1.2 µL of RNase-free water, 0.2 µL of QuantiNova Probe RT-Mix, 1.6 µL each of 10 µM forward and reverse primer, 0.4 µL of 10 µM probe, and 5 µL of extracted RNA as the template. Reactions were incubated at 45°C for 10 min for reverse transcription, followed by 95°C for 5 min for denaturation, and then subjected to 45 cycles of 95°C for 5 s and 55°C for 30 s. Signal detection and measurement were taken in each cycle after the annealing step. The cycling profile ended with a cooling step at 40°C for 30 s. The primers and probe sequences were against the RNA-dependent RNA polymerase/helicase (RdRP/Hel) gene region of SARS-CoV-2: forward primer: 5′-CGCATACAGTCTTRCAGGCT-3′; reverse primer: 5′-GTGTGATGTTGAWATGACATGGTC-3′; specific probe: 5′-FAMTTAAGATGTGGTGCTTGCATACGTAGAC-IABkFQ-3′. The viral load reduction assay experiments were performed in triplicate.

### Crystallization of SARS-CoV-2 M^pro^ in complex with H102

Concentrations of 5 mg/mL and 10 mg/mL M^pro^ (in a solution containing 20 mM Tris, 150 mM NaCl, 1 mM EDTA, and 1 mM TCEP [pH 7.8]) were incubated with 10 mM H102 at 1:10 vol ratio at room temperature for 2 h. The crystals were obtained by using the sitting-drop vapor diffusion method by mixing 1 µL of protein with 1 µL of reservoir solution and then equilibrating the mixture against 100 µL of the reservoir solution at 18°C. The initial crystallization screenings were carried out using commercially available kits. The complexes were crystallized in a solution containing 0.1 M MES monohydrate pH 6.0 and 20% (wt/vol) polyethylene glycol monomethyl ether 2,000.

### Data collection and structure determination

Diffraction data were collected at the Shanghai Synchrotron Radiation Facility (SSRF) BL17U (wavelength, 0.97918 Å). For data collection, the crystals were cryoprotected by briefly soaking in reservoir solution supplemented with 20% (vol/vol) glycerol before flash-cooling in liquid nitrogen. The data set was processed with HKL2000 software. The structure was determined by the molecular replacement method using Phaser with the previously reported structure (PDB: 7C6S). The atomic models were built using Coot and refined with phenix.refine in Phenix, and the stereochemical qualities of the final models were assessed with MolProbity. Data collection, processing, and refinement statistics are summarized in Table S1.

### Statistical analysis

Each experiment was performed independently at least three times. Statistical analyses were conducted using GraphPad Prism (version 8.0). Error bars indicate standard deviations. *P*-values were calculated using Student’s *t-*test to compare each compound candidate with DMSO control. *P < 0.05.

## Data Availability

The coordinates and structure factors have been deposited in the PDB with accession code 8YSA. Additional data are provided in the supplemental material. Source data are provided with this paper.

## SUPPLEMENTAL MATERIAL

Supplemental material jvi.00658-25-s0001.pdf Fig. S1 and S2, Table S1, supplemental experimental procedures, and references.

ASM does not own the copyrights to Supplemental Material that may be linked to, or accessed through, an article. The authors have granted ASM a non-exclusive, world-wide license to publish the Supplemental Material files. Please contact the corresponding author directly for reuse.
