# Supplementary material for: Discovery of a potent covalent inhibitor that unusually distorts the catalytic dyad of SARS-CoV-2 main protease
Source: J Virol. 2025 Sep 15;99(10):e00658-25. doi: 10.1128/jvi.00658-25 (PMC12548382; doi:10.1128/jvi.00658-25)
Supplement: Supplemental material — Fig. S1 and S2, Table S1, supplemental experimental procedures, and references. [file jvi.00658-25-s0001.pdf]

Supplemental Material For

Discovery of a Potent Covalent Inhibitor That Unusually Distorts the Catalytic Dyad of SARS-CoV-2 Main Protease

Juan Wang[1,14], Xiaohong Sang[2,14], Wenyan Zheng[3,4,14], Jasper Fuk-Woo Chan[5,6,7,8,9,10,14], Jiao Zhou[2], Yan Xu[11,12], Pu Han[4], Yong Feng[4], Lifeng Fu[4], Jessica Oi-Ling Tsang[5,6], Shuofeng Yuan[5,6,7,8], Aaron Ciechanover[2,13], Jing An[12*], Kwok-Yung Yuen[5,6,7,8,9,10*], Jianxun Qi[4*], Ziwei Huang[1,2,11,12*]

[1] School of Life Sciences, Tsinghua University, Beijing, China.

[2] Ciechanover Institute of Precision and Regenerative Medicine, School of Medicine, The Chinese University of Hong Kong, Shenzhen, China.

[3] State Key Laboratory for Conservation and Utilization of Subtropical Agro-Bioresources, Guangxi University, Nanning, China.

[4] CAS Key Laboratory of Pathogen Microbiology and Immunology, Institute of Microbiology, Chinese Academy of Sciences, Beijing, China.

[5] State Key Laboratory of Emerging Infectious Diseases, Department of Microbiology, School of Clinical Medicine, Li Ka Shing Faculty of Medicine, The University of Hong Kong, Pokfulam, Hong Kong Special Administrative Region, China.

[6]InnoHK (Centre for Virology, Vaccinology and Therapeutics), 646266Hong Kong Science and Technology Park, Hong Kong Special Administrative Region, China

[7] Department of Infectious Diseases and Microbiology, The University of Hong Kong-Shenzhen Hospital, Shenzhen, Guangdong Province, China.

[8]Pandemic Research Alliance Unit, The University of Hong Kong, Hong Kong Special Administrative Region, China

[9]Guangzhou Laboratory, Guangzhou, Guangdong Province, China

[10]Department of Microbiology, Queen Mary Hospital, Pokfulam, Hong Kong Special Administrative Region, China

[11]The Chinese University of Hong Kong, Shenzhen Futian Biomedical Innovation R&D Center, Shenzhen, China

[12] Division of Infectious Diseases and Global Public Health, Department of Medicine,

School of Medicine, University of California at San Diego, La Jolla, CA, USA.
[13] Technion Rappaport Integrated Cancer Center, The Rappaport Faculty of Medicine and Research Institute, Technion-Israel Institute of Technology, Haifa, Israel.
[14] These authors contributed equally: Juan Wang, Xiaohong Sang, Wenyan Zheng, Jasper Fuk-Woo Chan.
[*]Corresponding authors: Jing An (jan@health.ucsd.edu); Kwok-Yung Yuen (kyyuen@hku.hk); Jianxun Qi (jxqi@im.ac.cn); Ziwei Huang (zhuang@health.ucsd.edu)

Table of Contents

I. Supporting Figure 1……..………………………………………..……......3
II. Supporting Figure 2……..………………………………………..……..…3
III. Supporting Table 1……..………………………………………..……..…..4
IV. Supporting experimental procedures……...……………………………...….5
V. ^{1}H NMR and ^{13}C NMR spectra of target compounds……………………….20
VI. Supporting References…………………..……………………………….28

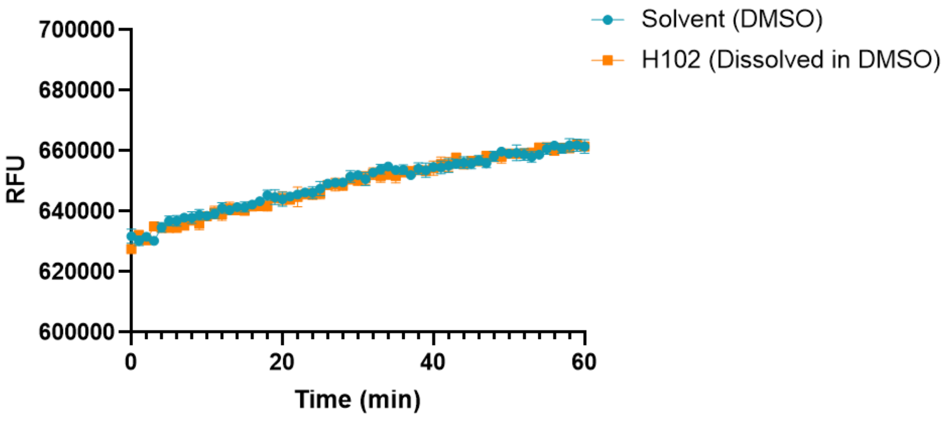

Supporting Figure 1. The fluorescence intensity of the Fmoc-Glu–EDANS solution in the presence or absence of 100 nM H102.

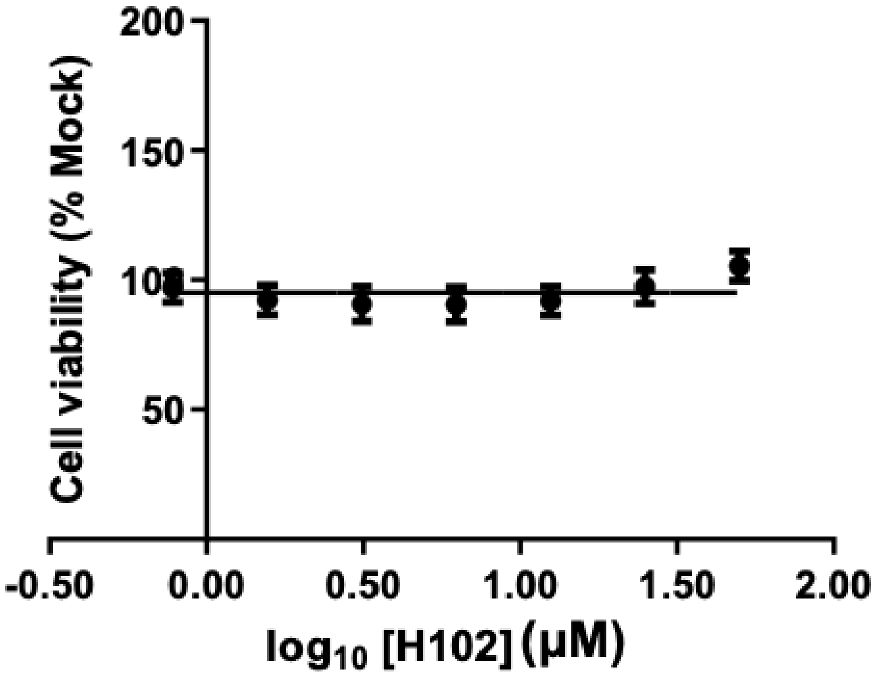

Supporting Figure 2. Cytotoxicity test of H102 on VeroE6 cells. The experiments were performed in triplicate, and the values are presented as mean ± SD.

Supporting Table 1. Diffraction data and refinement statistics

*Values in parentheses are for highest-resolution shell

	M^{pro} H102
**PDB Code**	8YSA
**Data collection**	
Space group	P622
Cell dimensions	
a, *b*, *c* (Å)	106.33, 106.33, 82.17
α, β, γ (°)	90, 90, 120
Wavelength (Å)	0.979
Resolution (Å)	50-1.50 (2.07-2.00)
R_{merge}	0.098 (1.066)
$I / \sigma I$	145.4 / 3.0 (9.6 / 2.2)
CC1/2	0.999 (0.910)
Completeness (%)	100 (100.0)
Redundancy	37.9 (31.3)
**Refinement**	
Resolution (Å)	26.56-2.00
No. reflections	44165
R_{work} / R_{free}	0.1811/0.1967
No. atoms	
Protein	2361
Ligand/ion	37
Water	291
B-factors	
Protein	19.5
Ligand/ion	15.8
Water	32.0
R.m.s. deviations	
Bond lengths (Å)	0.004
Bond angles (°)	0.754
Ramachandran plot	
Favored (%)	97.68
Allowed (%)	2.32
Outliers (%)	0

Supporting experimental procedures

Synthesis of M^{pro} Inhibitors

The synthesis for α-ketoamide or aldehyde based intermediate and final compounds are shown in Supplementary Schemes 1-2. The intermediate **4** were prepared according to reported literature(1,2). Boc-L-Glu(OMe)-OMe **1** reacted with bromoacetonitrile to give compound **2** which was reduced and cyclized to afford compound **3**. The Boc group was removed using TFA to give intermediate **4.**

Target Compounds **H97-H99** and **H137** were prepared as described in Supplementary Scheme 1. Boc-Tyr(OMe)-OH first coupled with **4** to give **6.** It was reduced and then oxidated to **8,** followed by passerini reaction to give **9**. After Boc group was removed, **10** coupled with different Boc protected amino acid to afford **11**, which was then oxidated to target compounds.

Compound **H94**, **H96**, **H100-H102** were synthesized in an alternative pathway, as illustrated in Supplementary Scheme 2. The generalized synthetic route was similar as α-ketoamide proteasome inhibitor described previously(3). Amino acid methyl ester **5** or **12b-c** was coupled with Boc protected amino acid to afford **13a-c**. **13a-c** were hydrolyzed to give **14a-c**, followed by coupling with **4** to yield **15a-c**. Reduction with sodium borohydride afforded alcohols **16a-c**, which were subsequently oxidated into **17-18** and **H102** using 2-iodoxybenzoic acid. Passerini reaction with isonitrile afforded **19a-c**, with subsequent oxidation to target compounds **H94**, **H100** and **H101**.

Similarly, **H96** was prepared from coupling of 2-(4-acetylphenyl)thiazole-5-carboxylic acid **23** and intermediate **20**, followed by oxidation.

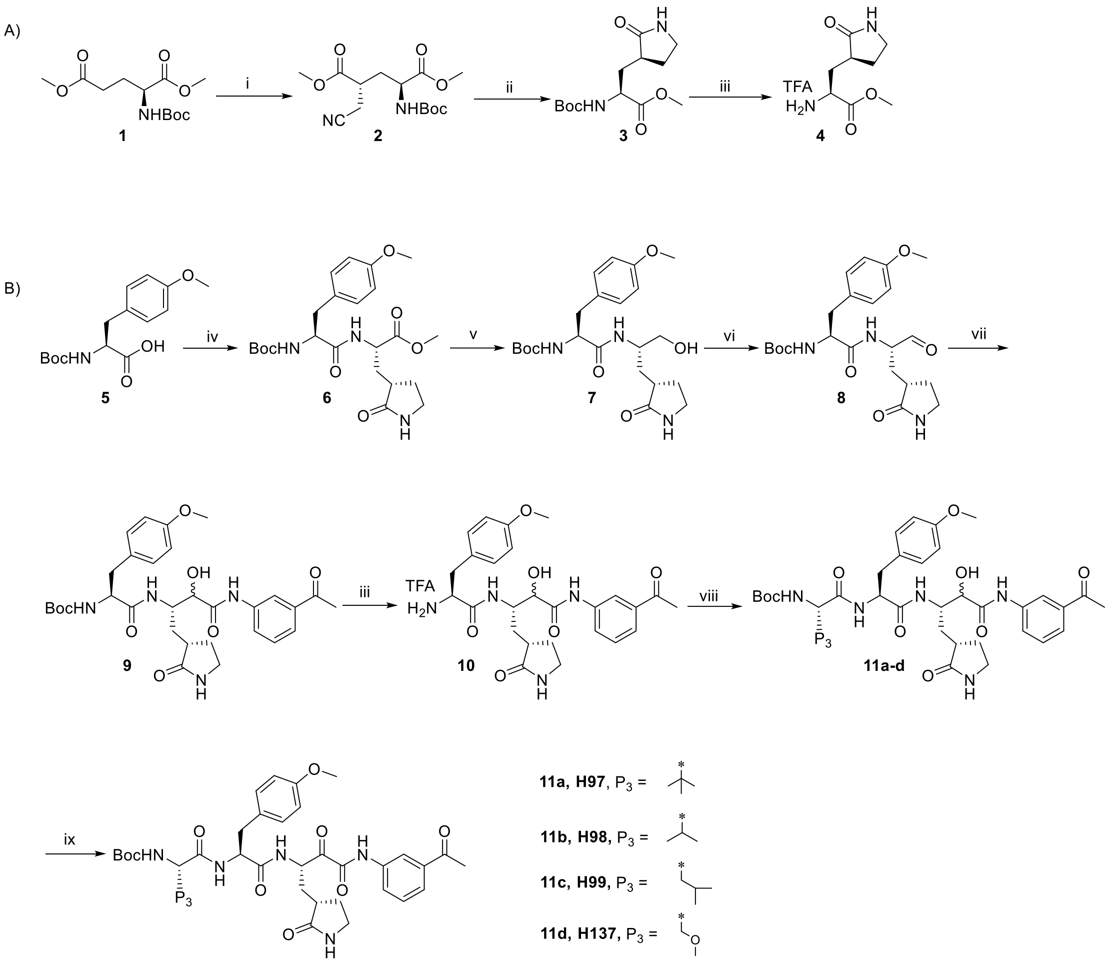

Supplementary Scheme 1. Synthetic route for compounds **H97-H99, H137**

[a]Reagents and conditions: (i) $BrCH_2CN$, 1M LiHMDS in THF, THF, -78 °C, 5 h; (ii) $CoCl_2.6H_2O$, CH_3OH, $NaBH_4$, 0 °C→ rt, 12 h; (iii) TFA, CH_2Cl_2, 0 °C→rt, 3 h; (iv) EDCI, HOBt, NMM, THF, 0 °C, 2 h; (v) $NaBH_4$, CH_3OH, 0 °C→rt, 5 h; (vi) 2-iodoxybenzoic acid, DMSO, rt, 4 h; (vii) substituted phenyl isonitrile, pyridine, TFA, dry CH_2Cl_2, -5 °C→0 °C, 3 h; (viii) Boc-AA-OH, EDCI, HOBt, TEA, ACN:DMF(4:1), 0 °C→rt, 1.5 h; (ix) 2-iodoxybenzoic acid, DMSO, rt, 4-5 h.

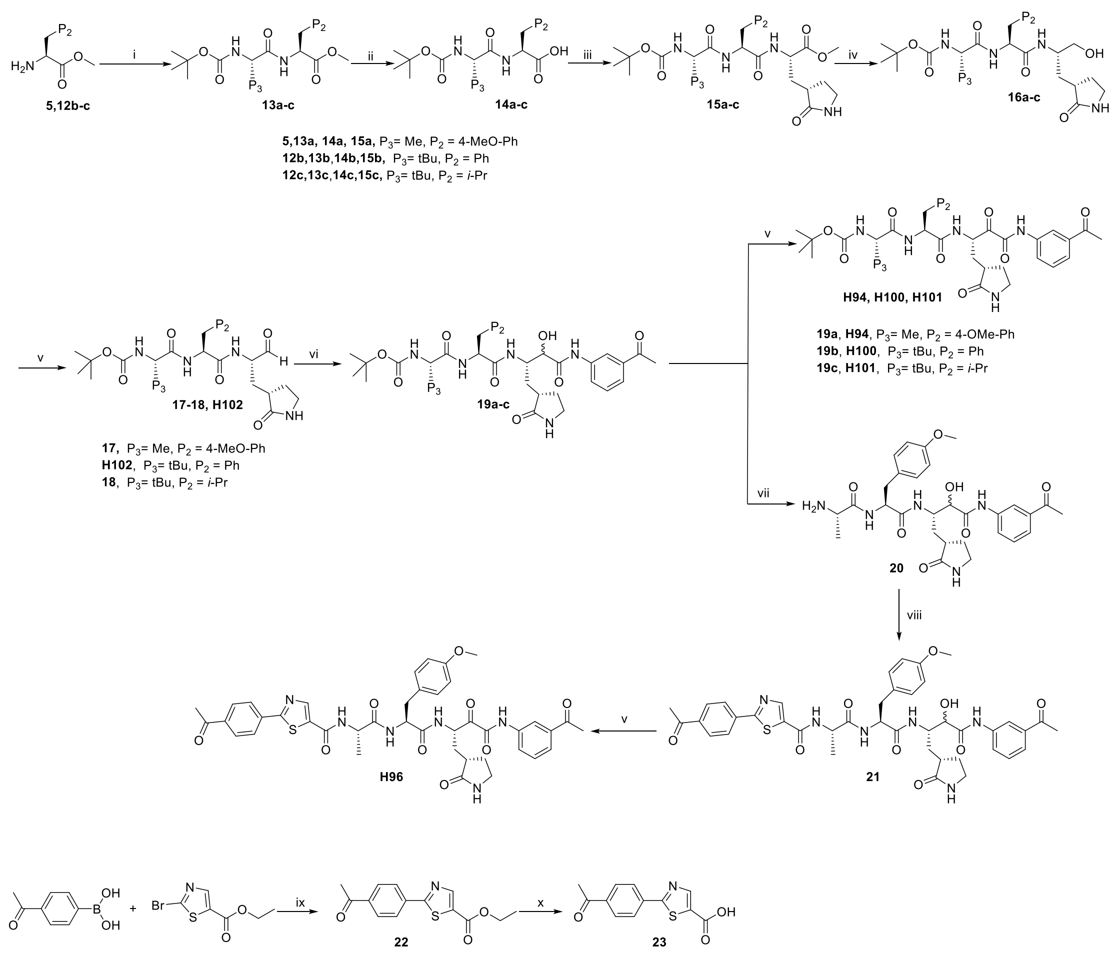

Supplementary Scheme 2. Synthetic route for compounds **H94, H96, H100-H102**

[a]Reagents and conditions: (i) Boc-AA-OH, HATU, HOBt, DIEA, THF, 0 °C, 1 h; (ii) LiOH.H_2O, THF, H_2O, 0 °C→ rt, 3 h; (iii) EDCI, HOBt, NMM, THF, 0 °C, 2h; (iv) $NaBH_4$, CH_3OH, 0 °C→rt, 5 h; (v) 2-iodoxybenzoic acid, DMSO, rt, 4-5 h; (vi) substituted phenyl isonitrile, pyridine, TFA, dry CH_2Cl_2, -5 °C→0 °C, 3 h; (vii) TFA, CH_2Cl_2, 0 °C→rt, 3 h; (viii) Intermediate **23**, EDCI, HOBt, TEA, ACN:DMF(4:1), 0 °C→rt, 1.5 h; (ix) Pd(dppf)Cl_2, K_2CO_3, dioxane, H_2O, 100 °C, 6 h; (x) LiOH.H_2O, THF, H_2O, rt, 3 h.

Experimental Details

Commercially available reagents and solvents were used directly without further purification. The high resolution MS (HRMS) of target compounds were analyzed by using Waters Xevo G2 QTof. ^1H NMR and ^{13}C NMR spectra were obtained on the Bruker Ascend™ 400. The chemical shifts (δ) are reported in parts per million (ppm) using suitable deuterated NMR solvents in reference to tetramethyl silane (TMS) at 0

ppm. Multiplicities are defined as follows: s (singlet), d (doublet), t (triplet), q (quartet), dd (doublet of doublets), and m (multiplets). The reaction was monitored by TLC (silica gel GF254) or HPLC. The compounds were purified by Flash chromatography packed with silica gel (silica gel 100-200 mesh or 200-300 mesh).

Dimethyl (2*S*,4*R*)-2-((*tert*-butoxycarbonyl)amino)-4-(cyanomethyl)pentanedioate (2)

The solution of *N*-Boc-*L*-glutamic acid dimethyl ester **1** (11.9 g, 43.2 mmol, 1.0 equiv) in dry THF (120 mL) was cooled to -78°C. Then 1M lithium bis(trimethylsilyl)amide (LiHMDS) in THF (94 mL, 2.0 equiv) was added dropwise at -78°C. The resulting solution was stirred at -78°C for 30 min. Bromoacetonitrile (6.2 g, 47.5 mmol, 1.2 equiv) in dry THF was added dropwise to the above solution under -78°C. The reaction solution was stirred at -78°C for 3 h. Then quenched with saturated NH_4Cl solution (60 mL) and allowed to warm to ambient temperature. The resulting mixture was extracted with EtOAc (2×). The combined organic layer was washed with brine, dried over Na_2SO_4, filtered, and concentrated under vacuum. The crude product was purified by silica gel column eluted with 15% EtOAc/petroleum ether → 25% EtOAc/ petroleum ether to afford compound **2** as light yellow oil (10.0 g, 73% yield). ^{1}H NMR (400 MHz, $CDCl_3$) δ 5.20-5.07 (m, 1H), 4.47-4.31 (m, 1H), 3.77 (s, 3H), 3.76 (s, 3H), 2.90-2.72 (m, 3H), 2.24-2.09 (m, 2H), 1.45 (s, 9H).

Methyl (*S*)-2-((*tert*-butoxycarbonyl)amino)-3-((*S*)-2-oxopyrrolidin-3-yl)propanoate (3)

Compound **2** (10.0 g, 31.8 mmol, 1.0 equiv) was dissolved in dry methanol (150mL). The reaction solution was cooled to 0 ℃. $CoCl_2 \cdot 6H_2O$ (4.5 g, 19 mmol, 0.6 equiv) was added, followed by the addition of $NaBH_4$ (8.5 g, 22 mmol, 7.0 equiv) portionwised. The reaction solution was stirred at room temperature overnight. Then quenched with saturated NH_4Cl solution, concentrated to remove methanol. The resulting mixture was extracted with EtOAc (3×). The combined organic layer was dried over Na_2SO_4, filtered, and concentrated under vacuum. The crude product was

purified by silica gel column eluted with 75% EtOAc/petroleum ether → 3% CH_3OH/CH_2Cl_2 to afford compound **3** as light yellow solid (5.6 g, 61% yield). ^{1}H NMR (400 MHz, $CDCl_3$) δ 6.43-6.15 (m, 1H), 5.59-5.43 (m, 1H), 4.39-4.26 (m, 1H), 3.74 (s, 3H), 3.40-3.30 (m, 2H), 2.53-2.41 (m, 2H), 2.21-2.06 (m, 1H), 1.93-1.80 (m, 2H), 1.44 (s, 9H).

Methyl (*S*)-2-amino-3-((*S*)-2-oxopyrrolidin-3-yl)propanoate (4)

To a solution of **3** (5.5 g) in CH_2Cl_2 (10 mL) was added TFA (8 mL) at 0 ℃. The solution was stirred at room temperature for 3 h. Then the solution was concentrated to remove TFA. Then ethyl ether was added and removed by means of dropper carefully so as not to disturb the residue. The residue was concentrated to give TFA salt of compound **4** as light yellow oil (8.1 g, 100% yield).

Procedure for the preparation of H97-99 and H137

Boc-Tyr(OMe)-OH (4.6 g, 15.6 mmol) was dissolved in THF (80 mL), cooled to 0 °C. Followed by the addition of EDCI (3.1 g, 16.3 mmol), HOBt (2.2 g, 16.3 mmol), compound **4 (**7.8 g, 14.8 mmol), NMM (6.0 g, 59.2 mmol). The reaction solution was stirred at 0 °C for 2 h. Ethyl acetate was added and washed with NH_4Cl solution. The aqueous layer was separated and extracted with ethyl acetate again. The combined organic layer was washed with brine, dried over anhydrous sodium sulfate, filtered, concentrated under vacuum. The crude product was purified by flash column chromatography (CH_2Cl_2: methanol = 30:1) to give **6** as light yellow oil (4.0 g, 58% yield). ^{1}H NMR (400 MHz, $CDCl_3$) δ 7.75-7.42 (m, 1H), 7.12 (d, *J* = 7.8 Hz, 2H), 6.81 (d, *J* = 7.8 Hz, 2H), 6.53-6.35 (m, 1H), 5.25-5.10 (m, 1H), 4.59-4.30 (m, 2H), 3.77 (s, 3H), 3.72 (s, 3H), 3.37- 3.25 (m, 2H), 3.09-2.91 (m, 2H), 2.53-2.33 (m, 2H), 2.21-2.10 (m, 1H), 1.90-1.72 (m, 2H), 1.39 (s, 9H).

Compound **6** (4.0 g, 8.6 mmol) was dissolved in anhydrous methanol. The reaction solution was cooled to 0 ℃. Then $NaBH_4$ (2.6 g, 68.8 mmol) was added in portions. After addition completed, the ice bath was removed and the reaction was stirred at room temperature for 3 h. The reaction was quenched by NH_4Cl solution at 0-5 ℃, extracted with ethyl acetate (2×). The combined organic phase was dried over sodium sulfate, filtered, and concentrated. The crude product was purified by flash column

chromatography on silica gel to give **7** (2.9 g, 77% yield) as off white solid. ^{1}H NMR (400 MHz, $CDCl_3$) δ 7.12 (d, J = 7.6 Hz, 2H), 6.82 (d, J = 7.5 Hz, 2H), 6.23-6.10 (s, 1H), 5.33 -5.18(m, 1H), 4.42-4.26 (m, 1H), 3.99-3.89 (m, 1H), 3.77 (s, 3H), 3.58-3.40 (m, 2H), 3.37-3.24 (m, 2H), 3.14 (s, 1H), 3.06-2.90 (m, 2H), 2.42-2.19 (m, 2H), 2.01-1.88 (m, 2H), 1.84-1.72 (m, 1H), 1.42 (s, 9H).

Compound **7** (2.8 g, 6.4 mmol) was dissolved in DMSO (25 mL), followed by the addition of IBX (2.88 g, 10.0 mmol). The reaction solution was stirred at room temperature for 5 h. Saturated $NaHCO_3$ solution was added and extracted with ethyl acetate (3 ×). The combined organic layer was washed with saturated $NaHCO_3$ solution, brine, dried over anhydrous sodium sulfate, filtered, concentrated under vacuum. The crude product was purified by flash column chromatography (CH_2Cl_2: methanol = 25:1) to give **8** (1.5 g, 54% yield).

To a solution of **8** (1.95 g, 4.5 mmol) and isonitrile (2.63 g, 18.0 mmol) in dry CH_2Cl_2, trifluoroacetic acid (1.03 g, 9.0 mmol) was added dropwise at -5 ℃. The reaction mixture was allowed to stir for 2 h at 0 ℃. Then pyridine (1.8 g, 22.5 mmol) was added at 0 ℃. The resulting solution was stirred at 0 ℃ for 2 h. Then concentrated under reduced pressure. The crude product was purified by flash column chromatography (CH_2Cl_2: methanol = 20:1) to give **9** as off white solid (1.6 g, 59% yield).

To a solution of **9** (1.6 g, 2.68 mmol) in CH_2Cl_2 (10 mL) was added TFA (8 mL) at 0 ℃ and stirred at 0 °C for 4 h. Then the solution was concentrated to remove TFA. Then ethyl ether was added to remove TFA. The residue was concentrated to give compound **10** as light yellow oil (1.7 g). ^{1}H NMR (400 MHz, DMSO-d_6) δ 9.94 (s, 1H), 8.31-8.21 (m, 2H), 7.98-7.93 (m, 1H), 7.92-7.87 (m, 2H), 7.70-7.57 (m, 2H), 7.43 (t, J = 7.9 Hz, 1H), 7.07 (d, J = 8.6 Hz, 2H), 6.78 (d, J = 8.6 Hz, 2H), 4.39-4.30 (m, 1H), 4.17-4.15 (m, 1H), 3.68 (s, 3H), 3.20 (t, J = 8.9 Hz, 1H), 3.15-3.06 (m, 1H), 2.99-2.91 (m, 1H), 2.59-2.53 (m, 1H), 2.48 (s, 3H), 2.33-2.20 (m, 2H), 2.17-2.06 (m, 1H), 1.77-1.64 (m, 1H), 1.46-1.35(m, 1H).

Boc protected amino acid (1.1 equiv) was dissolved in ACN/DMF (v:v = 4:1) and

cooled to 0 ℃. Followed by the addition of EDCI (1.1 equiv), HOBt (1.1 equiv), TFA salt of **10** (1.0 equiv), TEA (4.0 equiv). The resulting reaction solution was stirred at 0 ℃ for 1.5 h. Ethyl acetate was added and washed with NH_4Cl solution. The aqueous layer was separated and extracted with ethyl acetate again. The combined organic layer was washed with brine, dried over anhydrous sodium sulfate, filtered, concentrated under vacuum. The crude product was purified by flash column chromatography (CH_2Cl_2: methanol = 25:1) to give **11a-d.**

11a, off white solid, 92% yield. ^1H NMR (400 MHz, DMSO-d_6) δ 9.82 (s, 1H), 8.26 (d, *J* = 1.7 Hz, 1H), 8.01-7.94 (m, 1H), 7.88 (d, *J* = 7.9 Hz, 1H), 7.74-7.67 (m, 2H), 7.62 (d, *J* = 7.8 Hz, 1H), 7.57-7.51 (m, 1H), 7.45-7.39 (m, 2H), 7.04 (d, *J* = 8.4 Hz, 2H), 6.68 (d, *J* = 8.6 Hz, 2H), 6.30 (d, *J* = 9.7 Hz, 1H), 6.07 (d, *J* = 5.7 Hz, 1H), 4.57-4.48 (m, 1H), 4.30-4.21 (m, 1H), 4.11-4.06 (m, 1H), 3.82 (d, *J* = 9.9 Hz, 1H), 3.65 (s, 3H), 3.18-3.10 (m, 1H), 3.07-2.98 (m, 1H), 2.68-2.60 (m, 1H), 2.49 (s, 3H), 2.28-2.07 (m, 3H), 1.72-1.56 (m, 1H), 1.42-1.22 (m, 10H), 0.76 (s, 9H).

11b, off white solid, 76% yield. ^1H NMR (400 MHz, DMSO-d_6) δ 9.83 (s, 1H), 8.27 (s, 1H), 7.97 (d, *J* = 8.1 Hz, 1H), 7.86-7.70 (m, 2H), 7.63 (d, *J* = 7.7 Hz, 1H), 7.55 (s, 1H), 7.48-7.37 (m, 1H), 7.04 (d, *J* = 8.4 Hz, 2H), 6.68 (d, *J* = 8.7 Hz, 2H), 6.55-6.47 (m, 1H), 6.08 (d, *J* = 5.7 Hz, 1H), 4.58-4.48 (m, 1H), 4.30-4.21 (m, 1H), 4.12-4.07 (m, 1H), 3.66 (s, 3H), 3.19-3.11(m, 1H), 3.08-2.98 (m, 1H), 2.72-2.64 (m, 1H), 2.48-2.42(m, 1H), 2.30-2.03 (m, 3H), 1.85-1.74 (m, 1H), 1.70-1.58 (m, 1H), 1.35 (s, 9H), 1.26-1.20 (m, 1H), 0.76-0.63 (m, 6H).

11c, off white solid, 81% yield.

11d, light yellow solid, 84% yield. ^1H NMR (400 MHz, DMSO-d_6) δ 9.80 (s, 1H), 8.30 (s, 1H), 8.01-7.94 (m, 1H), 7.85 (d, J = 8.1 Hz, 1H), 7.74-7.62 (m, 2H), 7.59 -7.50 (m, 1H), 7.46-7.37 (m, 1H), 7.01 (d, *J* = 8.4 Hz, 1H), 6.80 (d, *J* = 8.1 Hz, 1H), 6.71-6.63 (m, 2H), 6.15 (d, *J* = 5.5 Hz, 1H), 4.53-4.43 (m, 1H), 4.30-4.18 (m, 1H), 4.16 -3.94 (m, 2H), 3.66 (s, 3H), 3.18 (s, 2H), 3.14-3.04 (m, 3H), 2.91-2.88 (m, 1H), 2.80-2.74 (m, 1H), 2.51 (s, 3H), 2.26-2.02 (m, 3H), 1.70-1.58 (m, 1H), 1.36 (s, 9H).

General Procedure A for the preparation of H97-99 and H137

11a-d (1.0 equiv) was dissolved in DMSO. Followed by the addition of IBX (1.8

equiv). The resulting solution was stirred at room temperature for 5 h. Saturated $NaHCO_3$ solution was added and extracted with ethyl acetate (3 ×). The combined organic layer was washed with saturated $NaHCO_3$ solution, brine, dried over anhydrous sodium sulfate, filtered, concentrated under vacuum. The crude product was purified by flash column chromatography (CH_2Cl_2: methanol = 30:1). Ethyl ether (100 mg/3 mL) was added to above obtained solid and was sonicated for 10 min. Then the solid was collected by filtration, and dried under vacuum to give final product **H97-99, H137.**

H97, white solid, 24% yield. ^{1}HNMR (400 MHz, $CDCl_3$) δ 9.22 (s, 1H), 8.31 (s, 1H), 8.23-8.14 (m, 1H), 7.99 (d, J = 7.9 Hz, 1H), 7.76-7.67 (m, 1H), 7.47-7.38 (m, 1H), 7.32-7.18 (m, 2H), 7.13-6.94 (m, 3H), 6.79-6.67 (m, 2H), 5.47-5.35(m, 1H), 5.25-5.13 (m, 1H), 4.91-4.74 (m, 1H), 3.83 (d, J = 9.4 Hz, 1H), 3.41-3.22 (m, 2H), 3.01-2.82 (m, 2H), 2.42-2.28 (m, 2H), 2.27-2.07 (m, 2H), 1.95-1.89(m, 1H), 1.43 (s, 9H), 0.90 (s, 9H). ^{13}C NMR (101 MHz, $CDCl_3$) δ 197.53, 195.00, 180.14, 171.09, 170.91, 158.39, 157.53, 155.91, 137.85, 137.05, 130.50, 129.41, 128.27, 124.99, 124.35, 119.75, 113.74, 79.90, 62.28, 55.14, 53.77, 53.36, 40.59, 37.55, 37.43, 34.29, 32.02, 28.31, 28.06, 26.67, 26.40. HRMS (ESI) calcd for $C_{37}H_{50}N_5O_9$ [M + H]$^+$ 708.3609, found 708.3602.

H98, white solid, 43% yield. ^{1}HNMR (400 MHz, $CDCl_3$) δ 9.12 (d, J = 29.3 Hz, 1H), 8.36-8.19 (m, 2H), 7.97 (d, J = 8.1 Hz, 1H), 7.71 (d, J = 8.0 Hz, 1H), 7.42 (t, J = 7.7 Hz, 1H), 7.08-6.98 (m, 2H), 6.79-6.65 (d, J = 7.9 Hz, 3H), 5.43-5.30 (m, 1H), 5.08-4.96 (m, 1H), 4.91-4.72 (m, 1H), 3.97-3.82 (m, 1H), 3.73 (s, 3H), 3.43-3.26 (m, 2H), 3.04-2.85 (m, 2H), 2.59 (s, 3H), 2.48-2.31 (m, 2H), 2.24-2.15(m, 1H), 2.06-1.85 (m, 1H), 1.50-1.37 (m, 9H), 0.91-0.73 (dd, J = 30.5, 6.2 Hz, 6H). ^{13}C NMR (101 MHz, $CDCl_3$) δ 197.51, 180.06, 171.59, 158.47, 157.54, 155.93, 137.89, 137.02, 130.49, 130.31, 129.43, 128.25, 124.99, 124.33, 119.73, 113.83, 60.13, 55.17, 53.78, 53.40, 40.67, 37.80, 37.48, 31.89, 30.68, 26.68, 19.22, 17.70. HRMS (ESI) calcd for $C_{36}H_{48}N_5O_9$ [M + H]$^+$ 694.3452, found 694.3448.

H99, white solid, 24% yield. ^{1}HNMR (400 MHz, $CDCl_3$) δ 8.95 (s, 1H), 8.28 (s, 2H), 7.95 (d, J = 8.1 Hz, 1H), 7.77 (d, J = 7.3 Hz, 1H), 7.48 (t, J = 7.7 Hz, 1H), 7.16-7.05 (m, 2H), 6.92-6.76 (m, 3H), 6.19-6.01 (m, 1H), 5.48-5.28 (m, 1H), 4.94-4.72 (m, 2H), 4.19-4.05 (m, 1H), 3.78 (s, 3H), 3.12-2.96 (m, 2H), 2.63 (s, 3H), 2.53-2.31(m, 2H),

2.15-2.03 (m, 2H), 2.00-1.87 (m, 1H), 1.49-1.38 (m, 9H), 0.96-0.83 (m, 6H). HRMS (ESI) calcd for $C_{37}H_{50}N_5O_9$ $[M + H]^+$ 708.3609, found 708.3598.

H137, white solid, 33% yield. ^1H NMR (400 MHz, $CDCl_3$) δ 9.06 (s, 1H), 8.31-8.16 (m, 2H), 7.93 (d, *J* = 7.9 Hz, 1H), 7.74 (d, *J* = 7.6 Hz, 1H), 7.46 (t, *J* = 7.7 Hz, 1H), 7.14-7.00 (m, 3H), 6.78 (d, *J* = 7.8 Hz, 2H), 6.28 (s, 1H), 5.51-5.23 (m, 2H), 4.87-4.73 (m, 1H), 4.31-4.11 (m, 1H), 3.74 (s, 3H), 3.72-3.64 (m, 1H), 3.52-3.45 (m, 1H), 3.41-3.33 (m, 2H), 3.30 (s, 3H), 3.09-2.99 (m, 2H), 2.61 (s, 3H), 2.52-2.37 (m, 2H), 2.17-2.03 (m, 2H), 2.01-1.95 (m, 1H), 1.42 (s, 9H). ^{13}C NMR (101 MHz, $CDCl_3$) δ 197.50, 194.74, 179.86, 171.10, 170.21, 158.51, 157.53, 137.95, 136.94, 130.52, 129.47, 128.23, 125.00, 124.33, 119.71, 113.86, 80.37, 71.88, 59.06, 55.18, 54.06, 53.33, 40.63, 37.12, 32.01, 28.42, 28.27, 26.71, 15.27. HRMS (ESI) calcd for $C_{35}H_{46}N_5O_{10}$ $[M + H]^+$ 696.3245, found 696.3234.

General Procedure B for the preparation of 13a-c

Boc protected amino acid (1.1 equiv) and TFA or HCl salt of **5, 12b-c**（1.0 equiv） was dissolved in THF (15 mL/g Boc protected amino acid). The resulting reaction solution was cooled to 0 °C, followed by the addition of HATU (1.1 equiv), HOBt (1.1 equiv), DIEA (3.5 equiv). The resulting solution was stirred at 0 °C for 1 h. After addition saturated aqueous NH_4Cl, the resulting mixture was extracted with ethyl acetate. The organic layer was separated, and washed with brine, dried over anhydrous sodium sulfate, filtered, concentrated under vacuum. The crude product was purified by silica gel column chromatography to afford **13a-c**.

Boc-Ala-Tyr(OMe)-OMe **13a**, colorless oil, 95% yield. ^1H NMR (400 MHz, $CDCl_3$) δ 7.01 (d, *J* = 8.6 Hz, 2H), 6.85- 6.79 (m, 2H), 6.47 (d, *J* = 7.6 Hz, 1H), 5.00-4.87(s, 1H), 4.84-4.75 (m, 1H), 4.18-4.12 (m, 1H), 3.78 (s, 3H), 3.72 (s, 3H), 3.16-2.97 (m, 2H), 1.44 (s, 9H), 1.32 (d, *J* = 7.0 Hz, 3H).

Boc-Tle-Phe-OMe **13b**, colorless oil, 97% yield.^1H NMR (400 MHz, $CDCl_3$) δ 7.32-7.27 (m, 2H), 7.26-7.20 (m, 1H), 7.12-7.06 (m, 2H), 6.08 (d, *J* = 7.3 Hz, 1H), 5.22 (d, *J* = 9.2 Hz, 1H), 4.91-4.80(m, 1H), 3.81 (d, *J* = 9.3 Hz, 1H), 3.72 (s, 3H), 3.19-3.03 (m, 2H), 1.45 (s, 9H), 0.95 (s, 9H).

Boc-Tle-Leu-OMe **13c**, colorless oil, 98% yield. ^1H NMR (400 MHz, $CDCl_3$) δ 6.08

(d, J = 7.8 Hz, 1H), 5.26 (d, J = 9.4 Hz, 1H), 4.67-4.56 (m, 1H), 3.85 (d, J = 9.5 Hz, 1H), 3.73 (s, 3H), 1.70-1.51 (m, 3H), 1.44 (s, 9H), 1.02 (s, 9H), 0.93 (d, J = 6.0 Hz, 6H).

General Procedure C for the preparation of 14a-c

Compound **13a-c** (1.0 equiv) was dissolved in THF/H_2O(v:v = 4:1). The reaction solution was cooled to 0 °C. LiOH.H_2O (2.0 equiv) was added. The reaction solution was stirred for 2 h at room temperature. THF was removed under reduced pressure. The residue was diluted with water, then adjusted pH to 2-3 with 1N HCl. The resulting mixture was extracted with ethyl acetate (2×). The organic layers were combined, washed with brine (2×), dried and concentrated under vacuum to give compound **14a-c**, used directly for the next step.

Boc-Ala-Tyr(OMe)-OH **14a**, off white solid, 98% yield. ^{1}H NMR (400 MHz, DMSO-d_6) δ 7.80 (d, J = 7.6 Hz, 1H), 7.11 (d, J = 8.5 Hz, 2H), 6.93 (d, J = 7.8 Hz, 1H), 6.85-6.75 (m, 2H), 4.40-4.27 (m, 1H), 4.00-3.86 (m, 1H), 3.70 (s, 3H), 3.04 -2.92 (m, 1H), 2.89-2.77 (m, 1H), 1.36 (s, 9H), 1.13 (d, J = 7.1 Hz, 3H).

Boc-Tle-Phe-OH **14b**, off white solid, 100% yield. ^{1}H NMR (400 MHz, DMSO-d_6) δ 12.66 (s, 1H), 8.13 (d, J = 7.7 Hz, 1H), 7.31-7.09 (m, 5H), 6.36 (d, J = 9.8 Hz, 1H), 4.52-4.40 (m, 1H), 3.88 (d, J = 9.8 Hz, 1H), 3.10-3.01 (m, 1H), 2.94-2.83 (m, 1H), 1.38 (s, 9H), 0.83 (s, 9H).

Boc-Tle-Leu-OH **14c**, off white solid, 100% yield.

General Procedure D for the preparation of 15a-c

Compound **14a-c** (1.2 equiv) was dissolved in anhydrous THF. The resulting solution was cooled to 0 ℃, followed by the addition of EDCI (1.1 equiv), HOBt (1.1 equiv), compound **4** (1.0 equiv), NMM (4.0 equiv). The reaction solution was stirred at 0 ℃ for 2h. After reaction completed, ethyl acetate was added, then washed with 0.1M HCl solution. The organic phased was separated, aqueous was extracted with ethyl acetate again. The organic layers were combined, washed with brine, dried over anhydrous sodium sulfate and concentrated under vacuum. The crude product was purified by silica gel column chromatography to afford **15a-c**.

15a, off white solid, 62% yield. ^{1}H NMR (400 MHz, $CDCl_3$) δ 7.53 (s, 1H), 7.12 (d, J

= 7.3 Hz, 2H), 6.89-6.74 (m, 3H), 5.91-5.76 (m, 1H), 5.13-5.01 (m, 1H), 4.74-4.60 (m, 1H), 4.22-4.08 (m, 1H), 3.95-3.85 (m, 1H), 3.80-3.75 (m, 3H), 3.58-3.44 (m, 2H), 3.38-3.27 (m, 2H), 3.22- 3.10 (m, 2H), 2.99-2.91(m, 1H), 2.43-2.31 (m, 1H), 2.23- 2.12 (m, 1H), 1.95-1.76 (m, 2H), 1.43 (d, *J* = 9.1 Hz, 9H), 1.33 (d, *J* = 6.8 Hz, 3H).

15b, off white solid, 64% yield. ^{1}H NMR (400 MHz, DMSO-d_6) δ 8.52 (d, *J* = 7.9 Hz, 1H), 8.02 (d, *J* = 7.8 Hz, 1H), 7.60 (s, 1H), 7.31-7.08 (m, 5H), 6.39 (d, *J* = 9.6 Hz, 1H), 4.64-4.56 (m, 1H), 4.67-4.54 (m, 1H), 4.24-4.37 (m, 1H), 3.61(s, 3H), 3.20-3.01 (m, 2H), 3.00-2.91 (m, 1H), 2.84-2.75 (m, 1H), 2.32-2.18 (m, 1H), 2.14-1.98 (m, 2H), 1.67-1.47 (m, 2H), 1.37 (s, 9H), 0.79 (s, 9H).

15c, off white solid, 98% yield. ^{1}H NMR (400 MHz, $CDCl_3$) δ 7.90-7.65 (m, 1H), 7.25-6.94 (m, 1H), 6.76-6.40 (m, 1H), 5.34-5.17 (m, 1H), 4.80-4.40 (m, 2H), 3.96-3.64 (m, 3H), 3.44-3.24 (m, 1H), 2.61-2.37 (m, 3H), 2.27- 2.14 (m, 1H), 1.94-1.80 (m, 1H), 1.76-1.60 (m, 2H), 1.50-1.35 (m, 9H), 1.10-0.82 (m, 15H).

General Procedure E for the preparation of 16a-c

Compound **15a**, **15c** (1.0 equiv) was dissolved in anhydrous methanol, compound **15b** was dissolved in methanol/THF (v:v = 1:1). The reaction solution was cooled to 0 ℃. Then $NaBH_4$ (8.0 equiv) was added in portions. After addition completed, the ice bath was removed and the reaction was stirred at room temperature for 3 h. The reaction was quenched by NH_4Cl solution at 0-5 ℃, extracted with ethyl acetate (2×). The combined organic phase dried over sodium sulfate, filtered, and concentrated. The crude product was purified by flash column chromatography on silica gel to give **16a-c**, used directly in the next step.

General Procedure F for the preparation of 17-18, H102

Compound **16a-c** (1.0 equiv) was dissolved in DMSO, followed by the addition of IBX (1.6 equiv). The reaction solution was stirred at room temperature for 4h. Saturated $NaHCO_3$ solution was added and extracted with ethyl acetate (3 ×). The combined organic layer was washed with saturated $NaHCO_3$ solution, water, brine, dried over anhydrous sodium sulfate, filtered, concentrated under vacuum. The crude product was purified by flash column chromatography (CH_2Cl_2: methanol = 30:1→25:1) to give **17**, **18** and **H102**. **H102** was further purified by C18 reverse-phase flash.

17, white solid, 51% yield. ^{1}H NMR (400 MHz, $CDCl_3$) δ 7.14 (d, *J* = 7.4 Hz, 2H), 6.98-6.75 (m, 3H), 6.14-5.82 (m, 1H), 5.20-4.96 (m, 1H), 4.85-4.62 (m, 1H), 4.47- 3.91 (m, 3H), 3.79 (s, 3H), 3.36-3.26 (m, 2H), 3.22- 2.89 (m, 2H), 2.42-2.22 (m, 1H), 2.17-1.95 (m, 1H), 1.93-1.85 (m, 1H), 1.81-1.69 (m, 1H), 1.45 (s, 9H), 1.34 (d, *J* = 6.8 Hz, 3H).

H102, white solid, 45% yield. ^{1}H NMR (400 MHz, $CDCl_3$) δ 9.37 (d, *J* = 66.1 Hz, 1H), 7.86 (s, 1H), 7.28-7.15(m, 5H), 7.10-6.97(m, 1H), 6.60-6.35(m, 1H), 5.25-5.04(m, 1H), 4.96-4.74 (m, 1H), 4.35-4.10 (m, 1H), 3.94-3.70 (m, 1H), 3.42-3.22 (m, 2H), 3.21-3.01 (m, 2H), 2.40-2.08 (m, 2H), 1.81-1.71 (m, 2H), 1.44 (s, 9H), 0.95 (s, 9H). ^{13}C NMR (101 MHz, $CDCl_3$) δ 198.5, 178.8, 170.6, 169.8, 155.0, 135.3, 128.6, 127.4, 125.8, 79.0, 61.4, 56.0, 53.0, 49.7, 39.5, 37.5, 36.6, 33.3, 28.71, 27.3, 27.1, 25.4. HRMS (ESI) calcd for $C_{27}H_{44}N_4O_6$ $[M + H]^+$ 517.3026, found 517.3030.

18, white solid, 70% yield. ^{1}H NMR (400 MHz, $CDCl_3$) δ 9.56-9.44 (m, 1H), 8.17-8.00 (m, 1H), 6.84-6.65 (m, 1H), 6.55 (s, 1H), 5.40-5.12 (m, 1H), 4.69-4.52 (m, 1H), 4.48-4.43(m, 1H), 3.90-3.76 (m, 1H), 3.45-3.28 (m, 2H), 2.59-2.32 (m, 2H), 2.03-1.79 (m, 3H), 1.75-1.51 (m, 3H), 1.43 (s, 9H), 1.05-0.91 (m, 15H). ^{13}C NMR (101 MHz, $CDCl_3$) δ 199.2, 180.0, 173.2, 171.0, 156.1, 80.0, 62.5, 57.1, 51.8, 42.1, 40.6, 37.9, 34.3, 31.6, 29.9, 28.3, 26.5, 24.8, 22.9, 22.6, 21.9, 14.1. HRMS (ESI) calcd for $C_{24}H_{43}N_4O_6$ $[M + H]^+$ 483.3183, found 483.3171.

General Procedure G for the preparation of 19a-c

To a solution of **17-18** or **H102** (1.0 equiv) and isonitrile (4.0 equiv) in dry CH_2Cl_2, trifluoroacetic acid (2.0 equiv) was added dropwise at -5 ℃. The reaction mixture was allowed to stir for 2 h at 0 ℃. Then pyridine (5.0 equiv) was added at 0 ℃. The resulting solution was stirred at 0 ℃ for 2 h. Then concentrated under reduced pressure. The crude product was purified by flash column chromatography (CH_2Cl_2: methanol = 20:1) to give products 19a-c, used directly in the next step.

Preparation of H94, H100, H101

Target compound **H94**, **H100**, **H101** was synthesized following general procedure A using **19a-c** as starting material.

H94, white dolid, 42%yield.^{1}H NMR (400 MHz, DMSO-*d_6*) δ 10.82 (s, 1H), 8.78-

8.67 (m, 1H), 8.44 (s, 1H), 8.03 (d, *J* = 8.3 Hz, 1H), 7.83-7.65(m, 3H), 7.52 (t, *J* = 7.6 Hz, 1H), 7.18-7.08 (m, 2H), 6.91-6.71 (m, 3H), 5.21-5.03(m, 1H), 4.60-4.46 (m, 1H), 4.00-3.75 (m, 1H), 3.68 (s, 3H), 3.24-3.05 (m, 2H), 2.98-2.84(m, 1H), 2.80-2.65 (m, 1H), 2.57 (s, 3H), 2.38-2.14 (m, 2H), 2.09-1.94 (m, 1H), 1.77-1.62 (m, 2H), 1.42-1.14 (m, 9H), 1.06 (d, *J* = 6.4 Hz, 3H). HRMS (ESI) calcd for $C_{34}H_{44}N_5O_9$ $[M + H]^+$ 666.3139, found 666.3109.

H100, white solid, 43%yield. ^1H NMR (400 MHz, $CDCl_3$) δ 9.17 (s, 1H), 8.29 (s, 1H), 8.20-8.08 (m, 1H), 7.98 (d, *J* = 7.4 Hz, 1H), 7.69 (d, *J* = 6.9 Hz, 1H), 7.48-7.37 (m, 1H), 7.34-7.29 (m, 1H), 7.24-7.00 (m, 6H), 5.48-5.33 (m, 1H), 5.23-5.09 (m, 1H), 4.96-4.76 (m, 1H), 3.89-2.75 (m, 1H), 3.42-3.20 (m, 2H), 3.07-2.87 (m, 2H), 2.57 (s, 3H), 2.42-2.28 (m, 2H), 2.25-2.03 (m, 2H), 1.44 (s, 9H), 0.89 (s, 9H). ^{13}C NMR (101 MHz, $CDCl_3$) δ 197.5, 195.0, 171.0, 157.5, 155.9, 137.9, 137.0, 136.3, 129.6, 129.4, 128.3, 126.6, 125.0, 124.3, 119.7, 79.9, 65.9, 62.3, 53.6, 53.3, 40.6, 38.3, 37.5, 34.2, 32.1, 28.3, 28.0, 26.7, 26.4, 15.3. HRMS (ESI) calcd for $C_{36}H_{48}N_5O_8$ $[M + H]^+$ 678.3503, found 678.3497

H101 white solid, 35% yield. ^1H NMR (400 MHz, $CDCl_3$) δ 9.15 (s, 1H), 8.46-8.28 (m, 2H), 8.08-7.88 (m, 1H), 7.76 (d, *J* = 7.4 Hz, 1H), 7.47 (t, *J* = 7.9 Hz, 1H), 7.20-7.08(m, 1H), 7.04-6.88 (m, 1H), 5.50-5.34 (m, 1H), 5.28-5.11(m, 1H), 4.72-4.54 (m, 1H), 3.88-3.76 (m, 1H), 3.46-3.29 (m, 2H), 2.62 (s, 3H), 2.60-2.51 (m, 1H), 2.44-2.12 (m, 3H), 1.64-1.53 (m, 2H), 1.52-1.45(m, 1H), 1.42 (s, 9H), 0.96 (s, 9H), 0.87-0.73 (m, 6H). ^{13}C NMR (101 MHz, $CDCl_3$) δ 197.6, 195.3, 180.4, 172.4, 171.1, 157.7, 156.0, 137.9, 137.2, 129.4, 124.9, 124.4, 119.8, 99.9, 80.0, 62.4, 53.4, 51.3, 42.1, 40.7, 37.5, 34.2, 31.9, 31.6, 28.3, 27.9, 26.7, 26.5, 24.5, 22.8, 22.7, 21.8, 14.1. HRMS (ESI) calcd for $C_{30}H_{50}N_5O_8$ $[M + H]^+$ 644.3659, found 644.3666.

Procedure for the preparation of 23

A mixture of 4-acetylphenylboronic acid (715 mg, 4.36 mmol, 1.0 equiv), ethyl 2-bromothiazole-5-carboxylate (1.03g, 4.36 mmol, 1.0 equiv), K_2CO_3 (1.81g, 13.1 mmol, 3.0 equiv) , $Pd(dppf)Cl_2$ (319 mg, 0.436 mmol, 0.1 equiv) in dioxane (15 mL) and H_2O (1.5 mL) was degassed and stirred at 100 ℃ for 6 h under nitrogen atmosphere. Then concentrated and purified by flash column chromatography (ethyl acetate:

petroleum ether = 100:15) to give **22** as off white solid (631 mg, 53% yield). ^{1}H NMR (400 MHz, $CDCl_3$) δ 8.47 (s, 1H), 8.12-8.02 (m, 4H), 4.41 (q, J = 7.1 Hz, 2H), 2.66 (s, 3H), 1.42 (t, J = 7.1 Hz, 3H).

Compound **22** (384 mg, 1.39 mmol, 1.0 equiv) was dissolved in THF (4mL), methanol (4mL), H_2O (1.2 mL). LiOH.H_2O (83.5mg, 3.49 mmol, 2.5 equiv) was added. The reaction solution was stirred for 4 h at room temperature. THF was removed under reduced pressure. The residue was diluted with water, then adjusted pH to 2-3 with 1N HCl. Compoud was collected by filteration and dried under vacuum at 50 °C to give **23** as white solid (236 mg, 69% yield).

Procedure for the preparation of H96

To a solution of **19a** (435 mg, 0.651 mmol) in CH_2Cl_2 (8 mL) was added TFA (8 mL) at 0 ℃. The solution was stirred at room temperature for 3 h. Then the solution was concentrated to remove TFA. Then ethyl ether was added to precipitate the product. The solid was collected by filtration to give compound **20** TFA salt as light yellow solid (441 mg).

Compound **23** (191 mg, 0.776 mmol) was dissolved in ACN (8 mL) and DMF (2 mL), cooled to 0 ℃. Followed by the addition of EDCI (131.6 mg, 0.686 mmol), HOBt (92.6 mg, 0.686 mmol), **20** TFA salt (407 mg, 0.597 mmol) and TEA (181 mg, 1.79 mmol). The reaction solution was stirred at 0 ℃ for 2h. Ethyl acetate was added and washed with 0.2N HCl solution, brine. The organic layer was separated, dried over anhydrous sodium sulfate, filtered, concentrated under vacuum. The crude product was purified by flash column chromatography (CH_2Cl_2: methanol = 15:1) to give **21** as off white solid (352 mg, 74% yield).^{1}H NMR (400 MHz, DMSO-d_6) δ 9.83 (s, 1H), 8.87 (d, J = 7.4 Hz, 1H), 8.61 (s, 1H), 8.30 (s, 1H), 8.20-8.02 (m, 4H), 7.97 (d, J = 8.0 Hz, 1H), 7.73 -7.48 (m, 3H), 7.46-7.36 (m, 1H), 7.04 (d, J = 7.7 Hz, 2H), 6.64 (d, J = 7.4 Hz, 2H), 6.17 (d, J = 5.2 Hz, 1H), 4.51-4.37 (m, 2H), 4.30-4.20 (m, 1H), 4.13-4.06 (m, 1H), 3.60 (s, 3H), 3.20 -3.12 (m, 1H), 2.80-2.70 (m, 1H), 2.63 (s, 3H), 2.29-2.03 (m, 3H), 1.71-1.60 (m, 1H), 1.40-1.29 (m, 1H), 1.28-1.22 (m, 3H).

Target compound **H96** was synthesized following general procedure A described

above using **21** as starting material. The crude product was purified by flash column chromatography (CH_2Cl_2: methanol = 25:1) to give 90 mg **H96**. The suspension of above product in CH_2Cl_2 (3 mL) was sonicated for 10 min. The solid was collected by filtration,and dried under vacuum to give **H96** (60 mg, 17% yield). ^{1}HNMR (400 MHz, DMSO-d_6) δ 10.82 (s, 1H), 8.87 (d, J = 7.4 Hz, 1H), 8.67 (d, J = 6.1 Hz, 1H), 8.61 (s, 1H), 8.45 (s, 1H), 8.21-7.98 (m, 6H), 7.78-7.67 (m, 2H), 7.52 (t, J = 7.8 Hz, 1H), 7.13 (d, J = 7.7 Hz, 2H), 6.71 (d, J = 7.4 Hz, 2H), 5.21-5.01 (m, 1H), 4.56-4.40 (m, 2H), 3.62 (s, 3H), 3.26-3.07(m, 2H), 3.01-2.88 (m, 1H), 2.81-2.71 (m, 1H), 2.63 (s, 3H), 2.57 (s, 3H), 2.39-2.16 (m, 2H), 2.10-1.94(m, 1H), 1.81-1.63 (m, 2H), 1.26 (d, J = 6.8 Hz, 3H).^{13}CNMR (101 MHz, DMSO-d_6) δ 197.98, 197.83, 196.23, 196.18, 178.54, 172.20, 171.98, 169.26, 159.98, 159.81, 158.19, 145.19, 138.61, 138.46, 137.73, 136.81, 136.65, 130.61, 129.82, 129.68, 127.13, 125.35, 124.91, 120.27, 113.85, 55.40, 55.23, 54.41, 52.50, 49.20, 38.03, 36.97, 31.69, 27.78, 27.38, 27.23, 17.99. HRMS (ESI) calcd for $C_{41}H_{43}N_6O_9S$ $[M + H]^+$ 795.2812, found 795.2827.

^{1}H NMR and^{13}C NMR spectra of target compounds

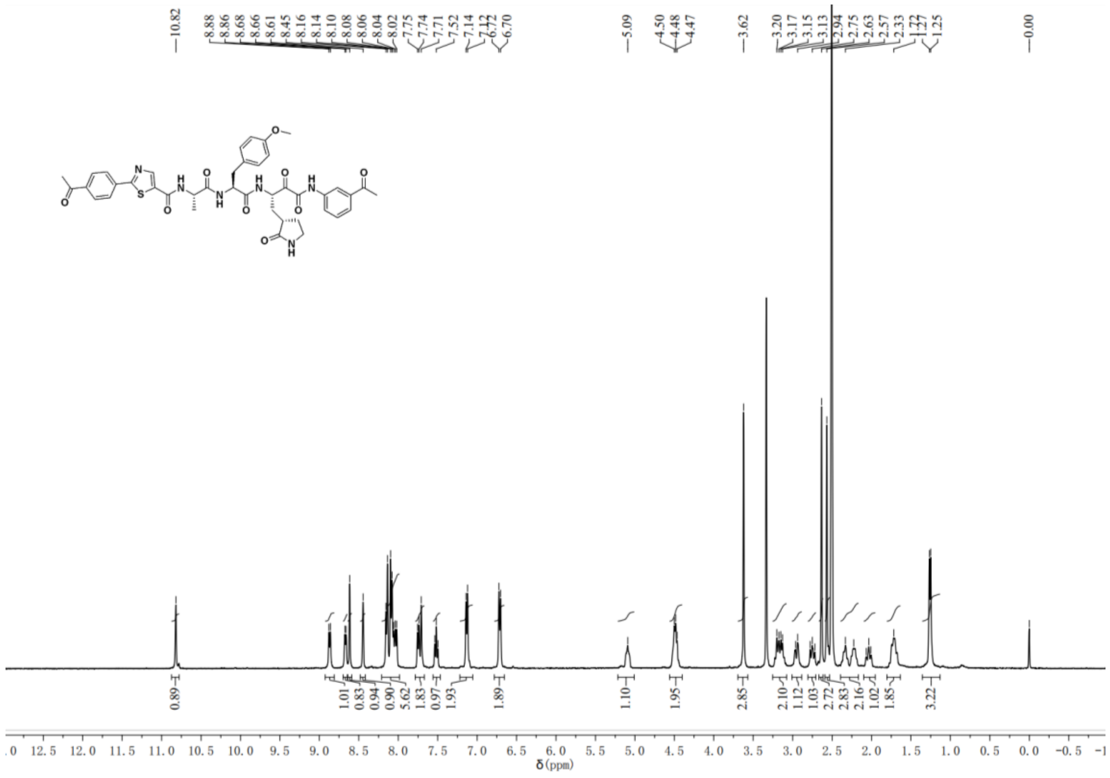

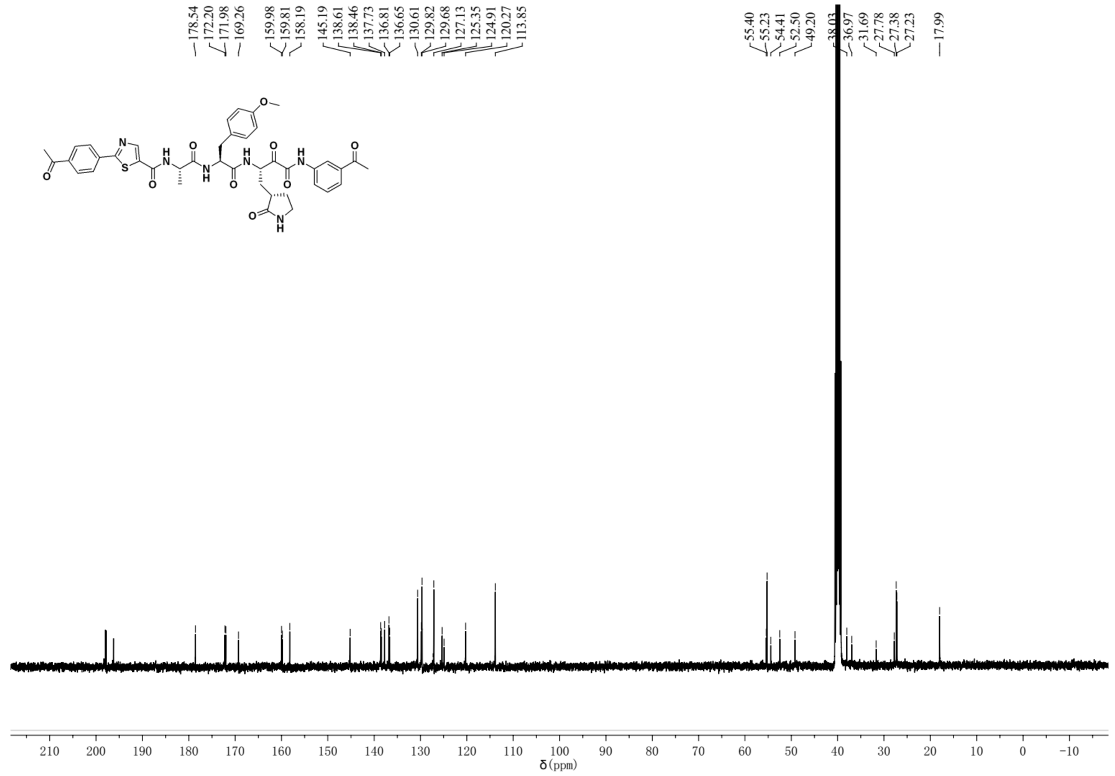

Supporting Figure 2. ^{1}H NMR and ^{13}C NMR of **H96**

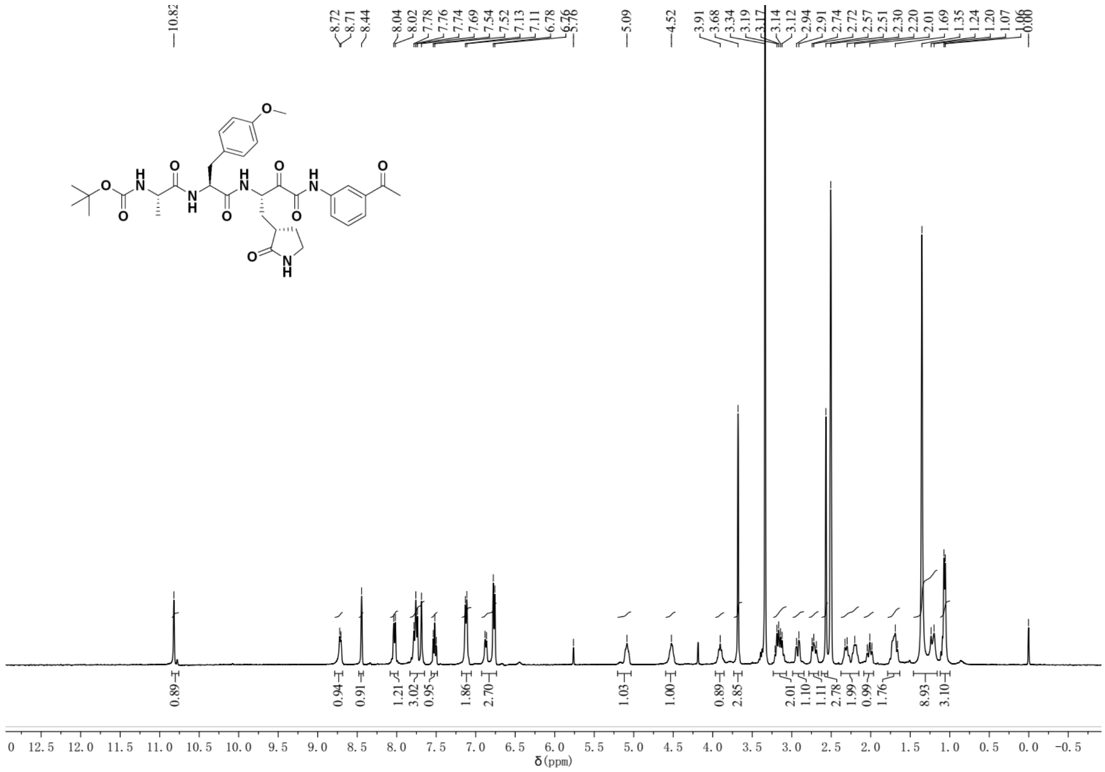

Supporting Figure 3. ^{1}H NMR of **H94**

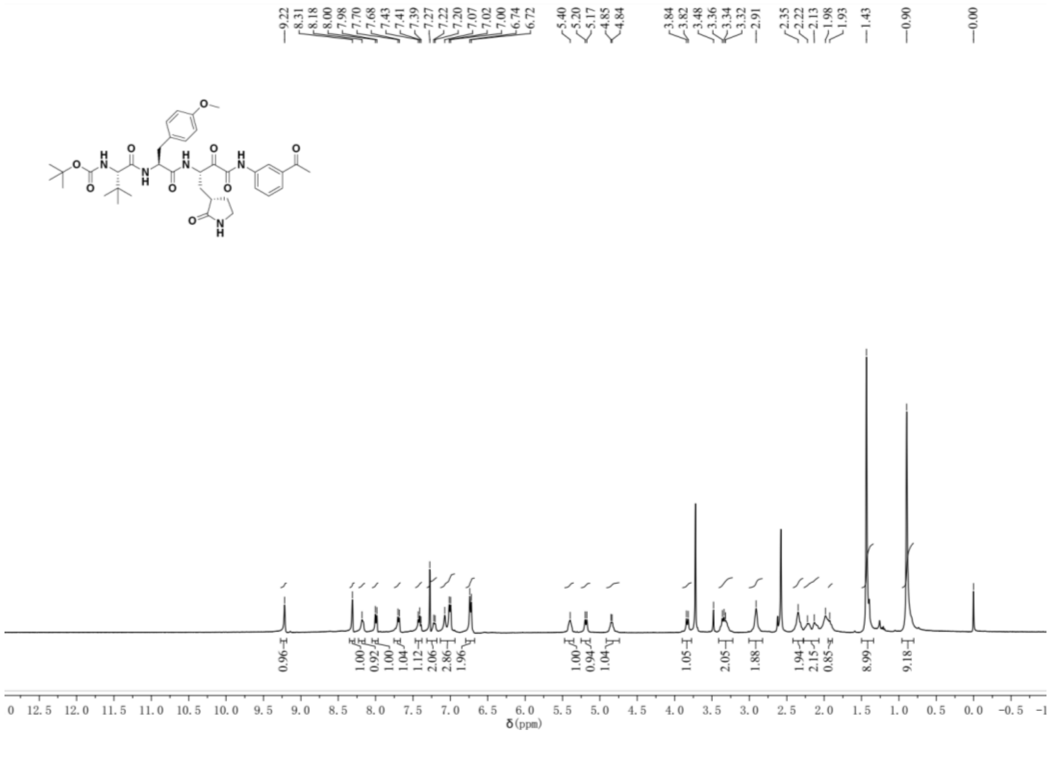

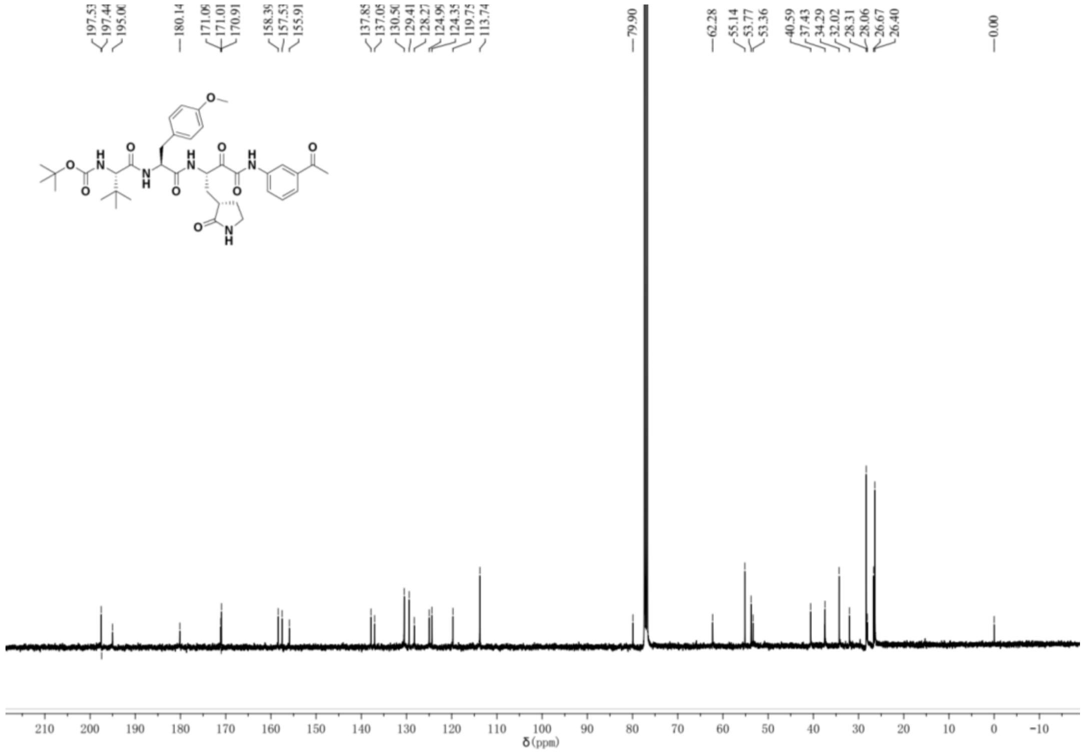

Supporting Figure 4. ^{1}H NMR and ^{13}C NMR of **H97**

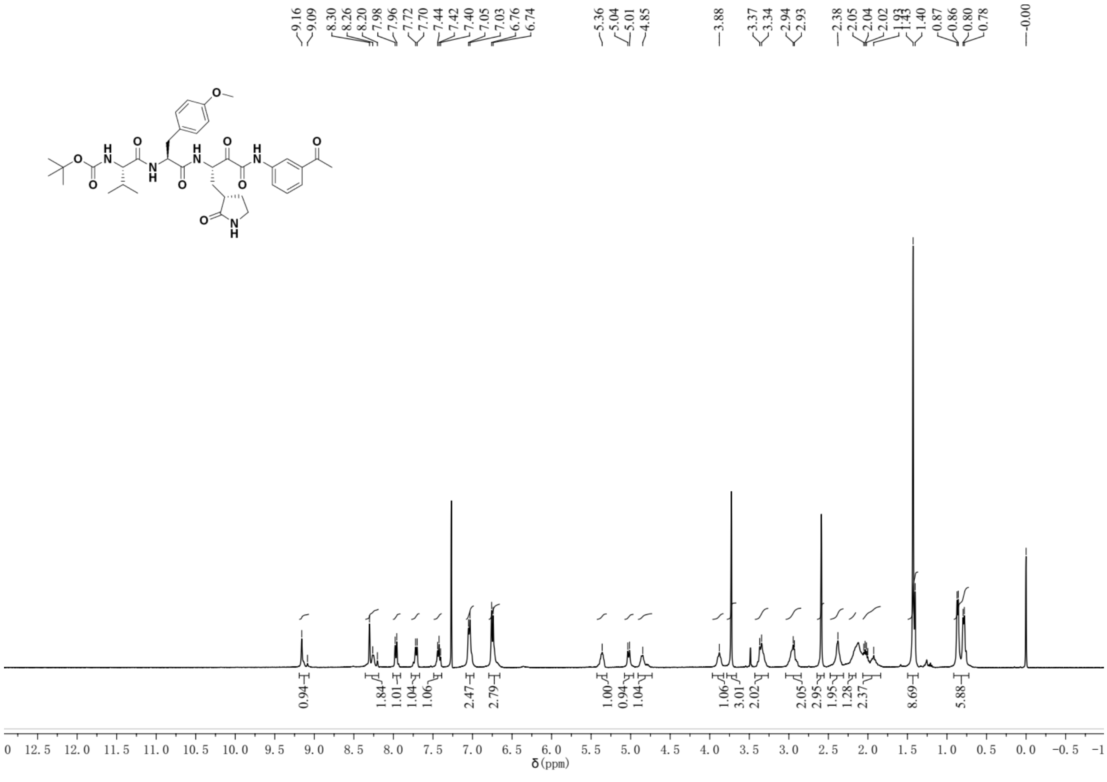

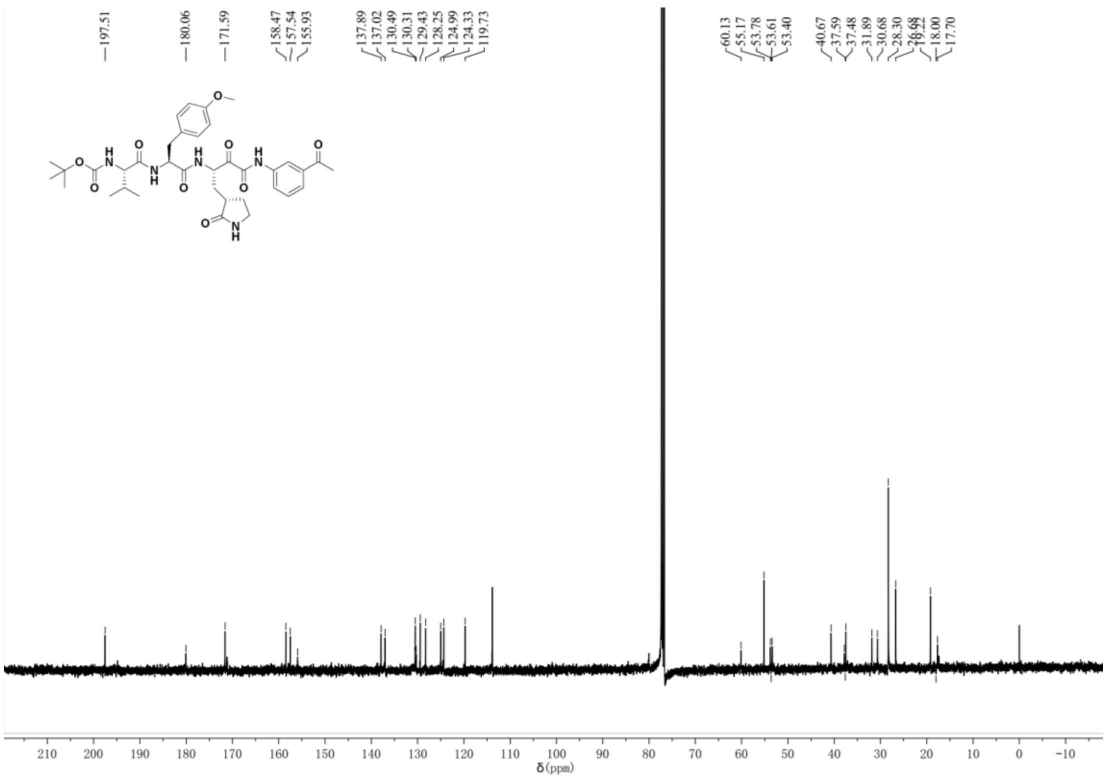

Supporting Figure 5. ^{1}H NMR and ^{13}C NMR of **H98**

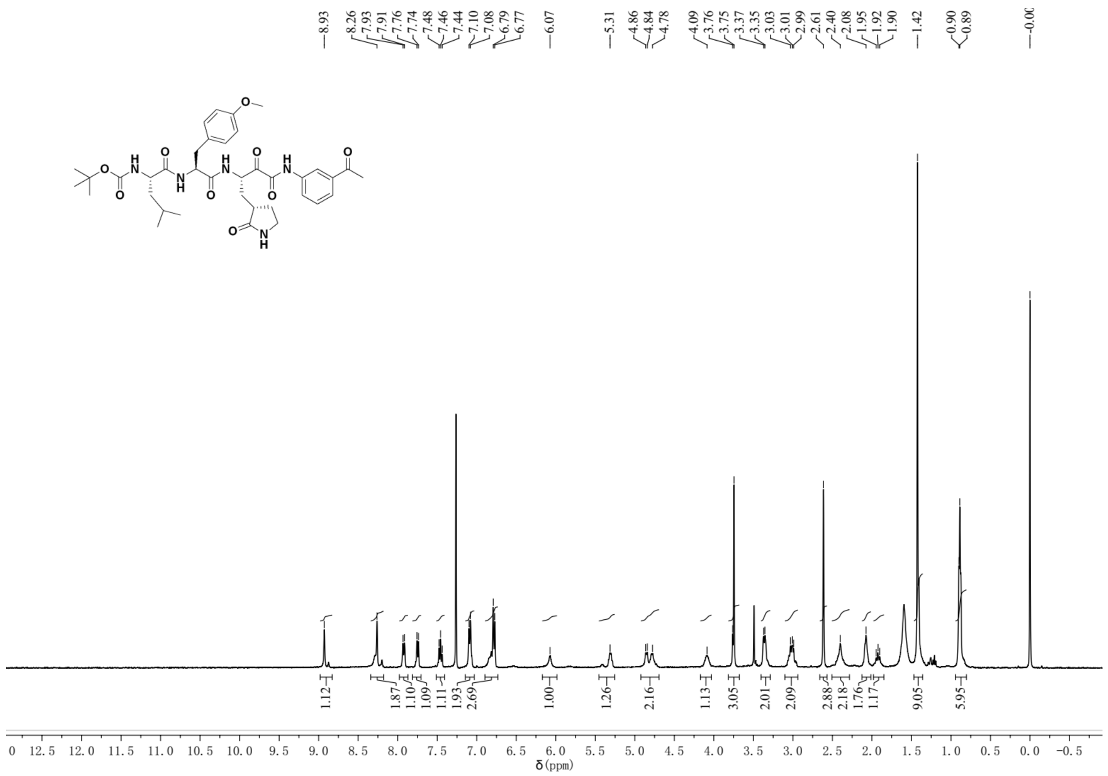

Supporting Figure 6. ^{1}H NMR of **H99**

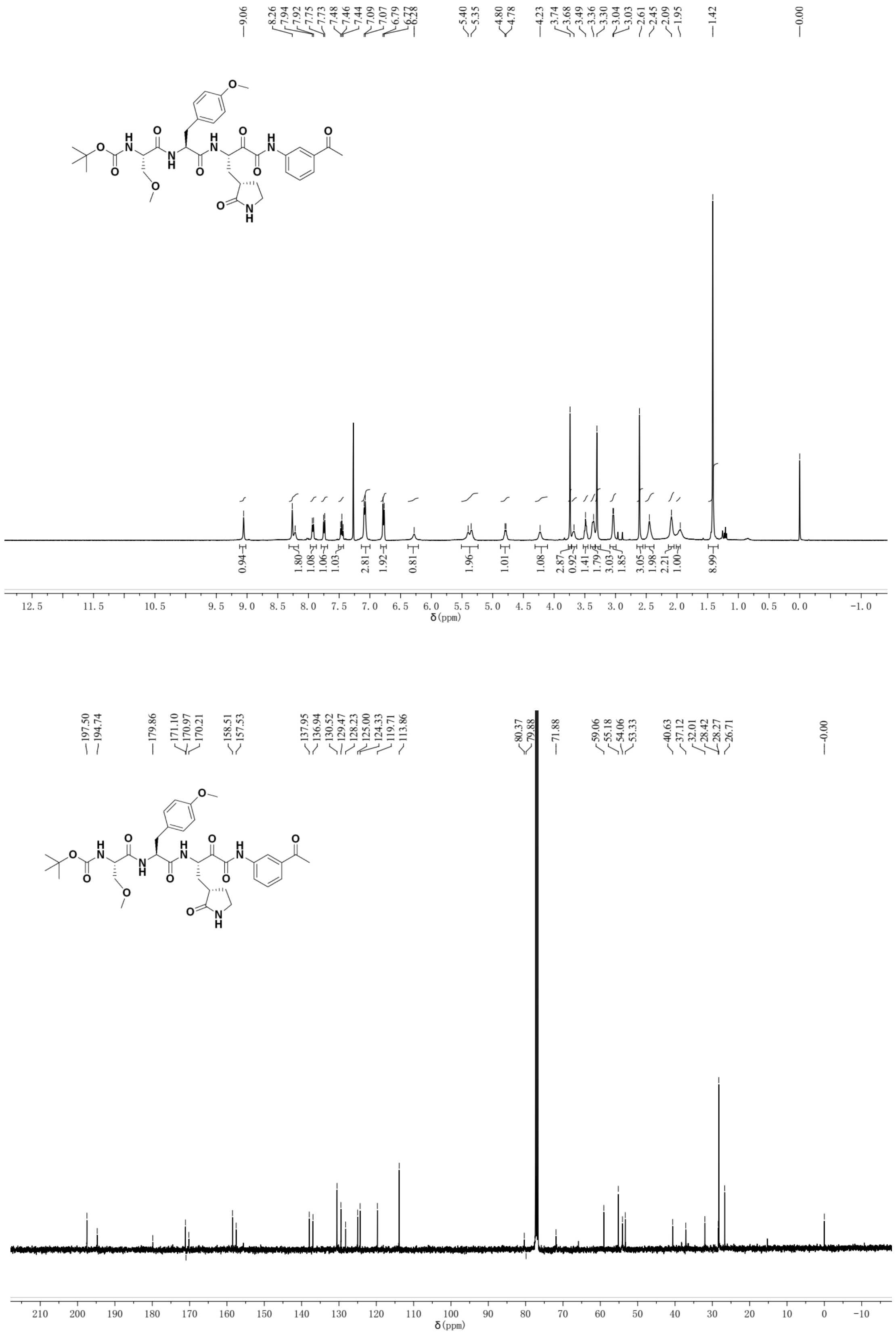

Supporting Figure 7. ^{1}H NMR and ^{13}C NMR of **H137**

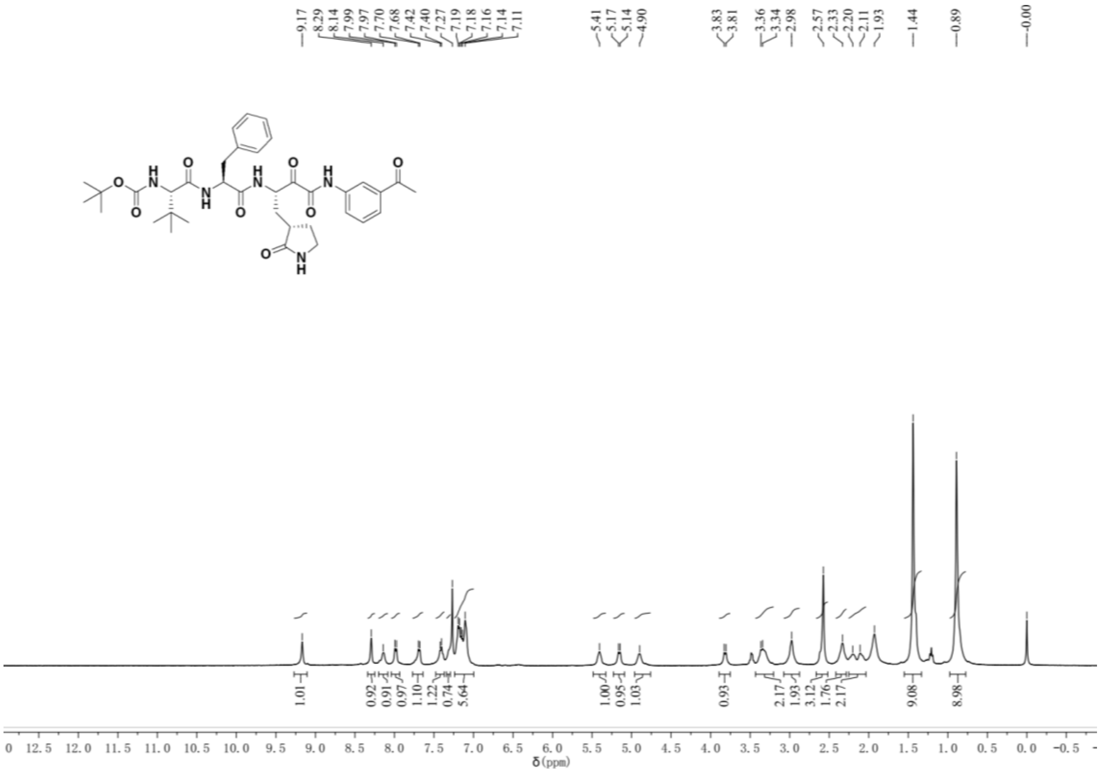

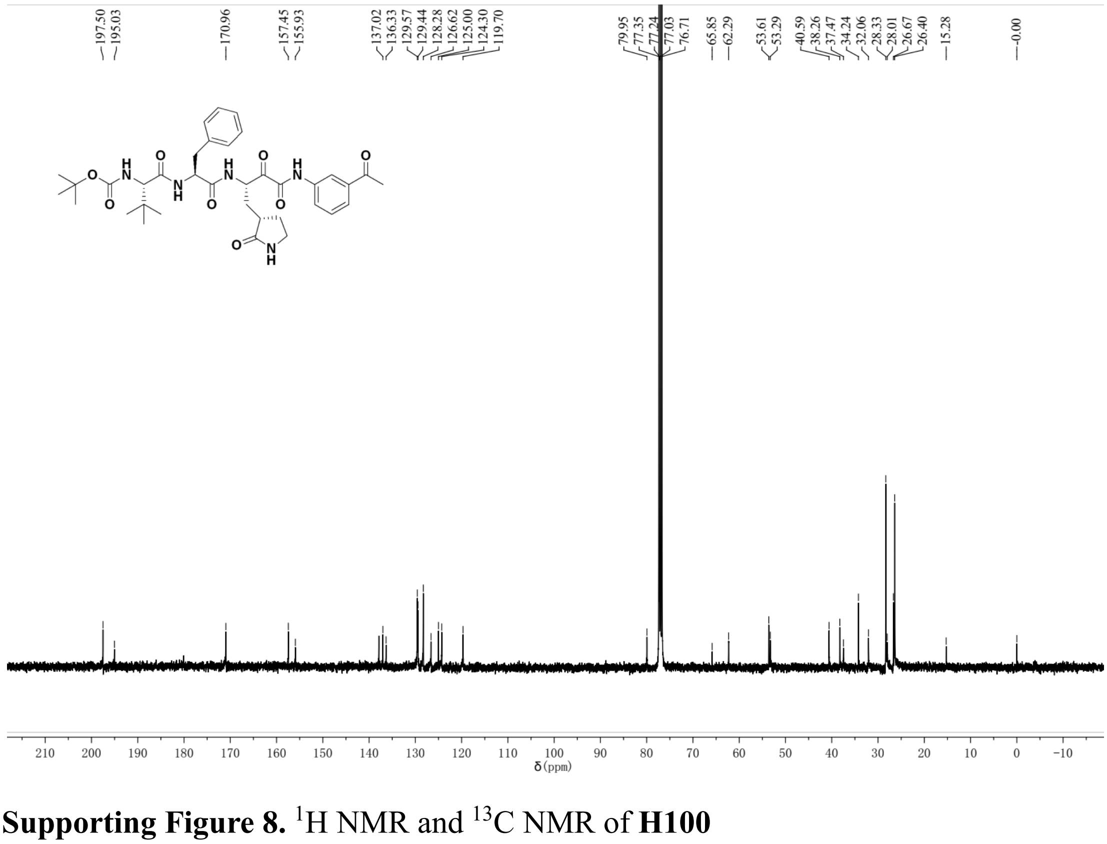

Supporting Figure 8. ^{1}H NMR and ^{13}C NMR of **H100**

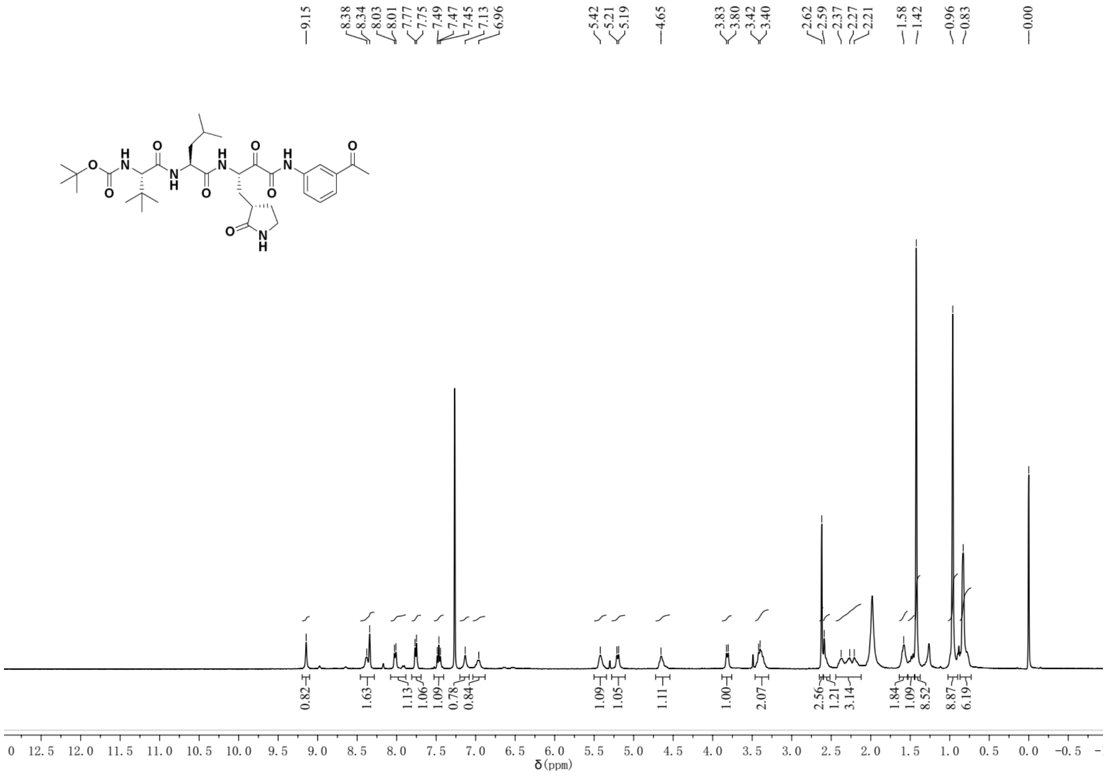

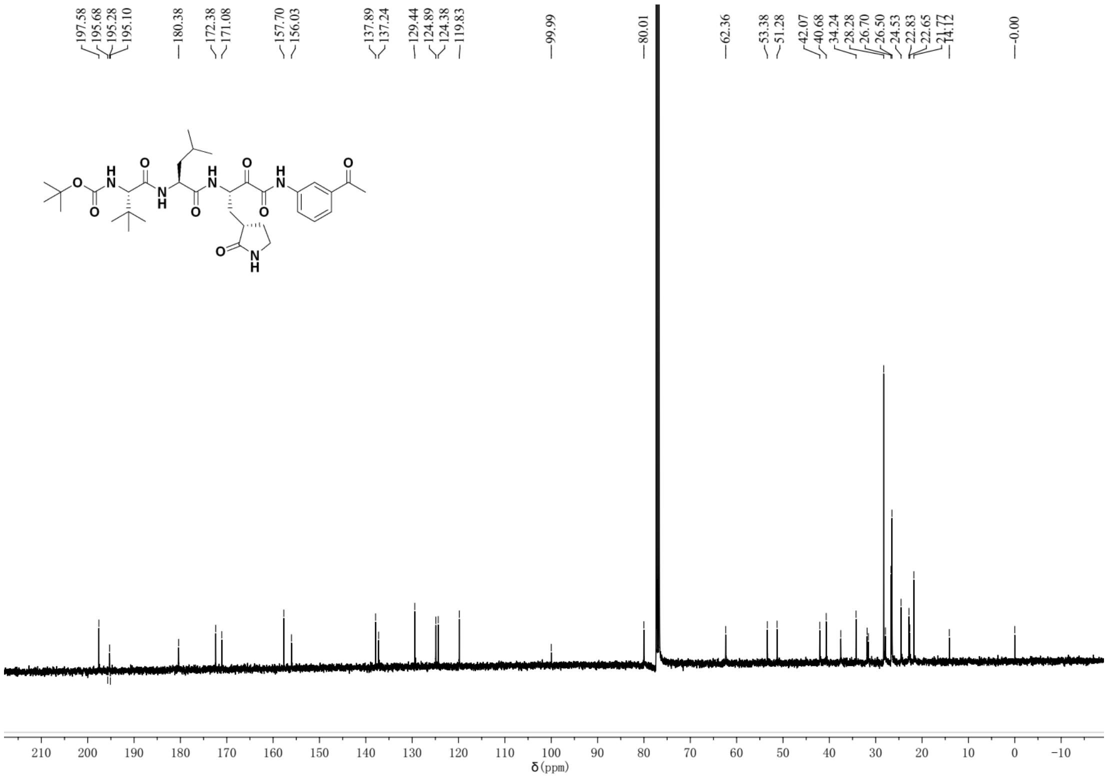

Supporting Figure 9. ^{1}H NMR and ^{13}C NMR of **H101**

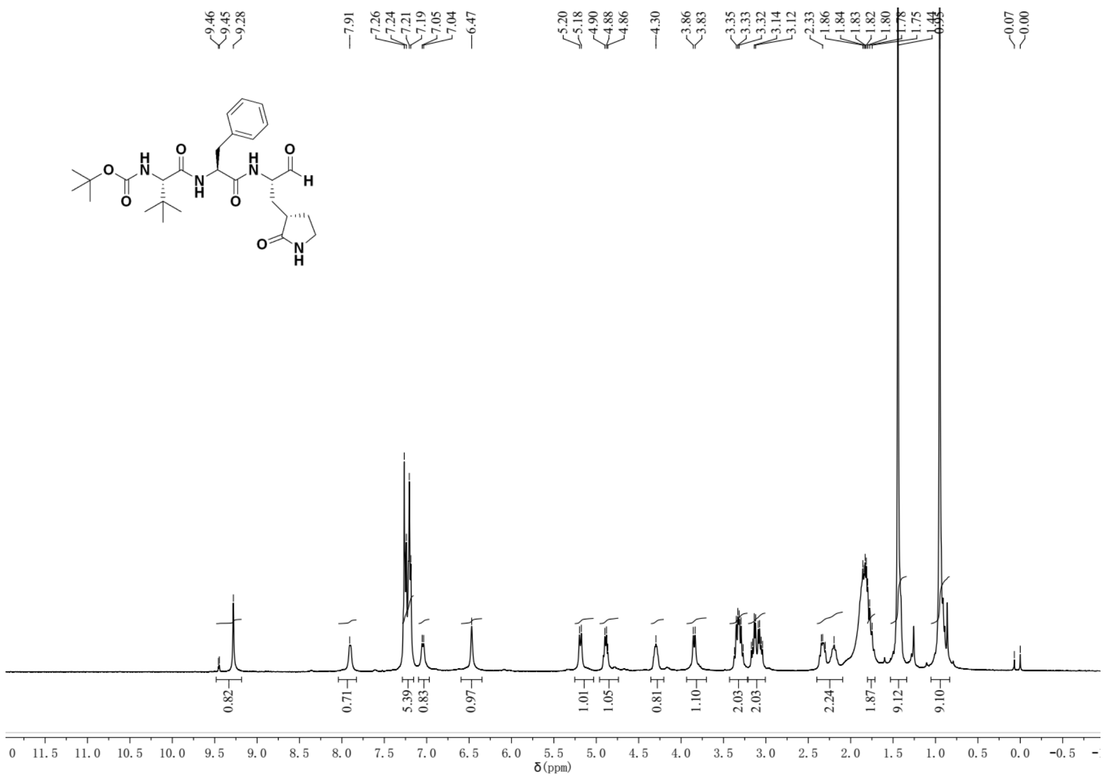

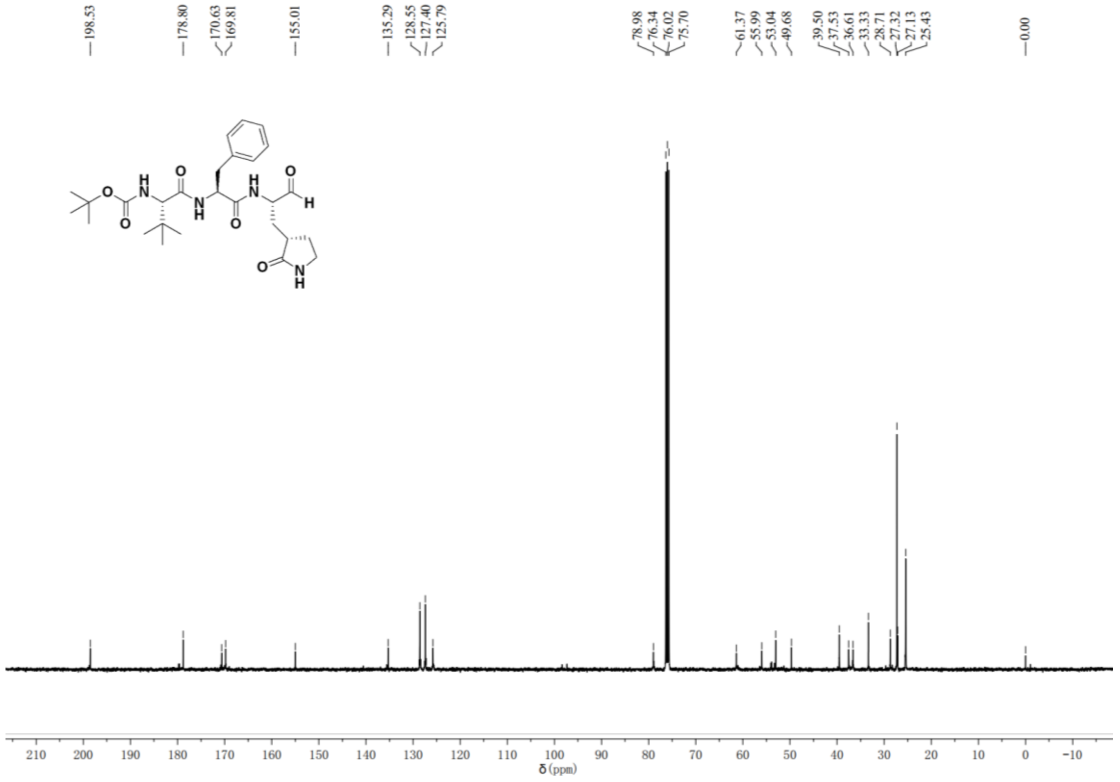

Supporting Figure 10. ^{1}H NMR and ^{13}C NMR of **H102**

Supporting References

1. Qiao, J., Li, Y.-S., Zeng, R., Liu, F.-L., Luo, R.-H., Huang, C., Wang, Y.-F., Zhang, J., Quan, B., Shen, C., Mao, X., Liu, X., Sun, W., Yang, W., Ni, X., Wang, K., Xu, L., Duan, Z.-L., Zou, Q.-C., Zhang, H.-L., Qu, W., Long, Y.-H.-P., Li, M.-H., Yang, R.-C., Liu, X., You, J., Zhou, Y., Yao, R., Li, W.-P., Liu, J.-M., Chen, P., Liu, Y., Lin, G.-F., Yang, X., Zou, J., Li, L., Hu, Y., Lu, G.-W., Li, W.-M., Wei, Y.-Q., Zheng, Y.-T., Lei, J., and Yang, S. (2021) SARS-CoV-2 M-pro inhibitors with antiviral activity in a transgenic mouse model. *Science* **371**, 1374-1378
2. Dai, W., Zhang, B., Jiang, X. M., Su, H., Li, J., Zhao, Y., Xie, X., Jin, Z., Peng, J., Liu, F., Li, C., Li, Y., Bai, F., Wang, H., Cheng, X., Cen, X., Hu, S., Yang, X., Wang, J., Liu, X., Xiao, G., Jiang, H., Rao, Z., Zhang, L. K., Xu, Y., Yang, H., and Liu, H. (2020) Structure-based design of antiviral drug candidates targeting the SARS-CoV-2 main protease. *Science* **368**, 1331-1335
3. Wang, J., Liang, B., Chen, Y., Chan, J. F.-W., Yuan, S., Ye, H., Nie, L., Zhou, J., Wu, Y., Wu, M., Huang, L. S., An, J., Warshel, A., Yuen, K.-Y., Ciechanover, A., Huang, Z., and Xu, Y. (2021) A new class of alpha-ketoamide derivatives with potent anticancer and anti-SARS-CoV-2 activities. *European Journal of Medicinal Chemistry* **215**
